# Comprehensive Analysis of m6A RNA Methylation Regulators in the Prognosis and Immune Microenvironment of Multiple Myeloma

**DOI:** 10.3389/fonc.2021.731957

**Published:** 2021-11-04

**Authors:** Rui Liu, Ying Shen, Jinsong Hu, Xiaman Wang, Dong Wu, Meng Zhai, Ju Bai, Aili He

**Affiliations:** ^1^ Department of Hematology, The Second Affiliated Hospital of Xi’an Jiaotong University, Xi’an, China; ^2^ Department of Cell Biology and Genetics, The Institute of Infection and Immunity, Xi’an Jiaotong University Health Science Center, Xi’an, China; ^3^ National–Local Joint Engineering Research Center of Biodiagnostics & Biotherapy, The Second Affiliated Hospital of Xi’an Jiaotong University, Xi’an, China

**Keywords:** multiple myeloma, m6A (N6-methyladenosine), RNA methylation, immune infiltration, survival

## Abstract

**Background:**

N6-methyladenosine is the most abundant RNA modification, which plays a prominent role in various biology processes, including tumorigenesis and immune regulation. Multiple myeloma (MM) is the second most frequent hematological malignancy.

**Materials and Methods:**

Twenty-two m6A RNA methylation regulators were analyzed between MM patients and normal samples. Kaplan–Meier survival analysis and least absolute shrinkage and selection operator (LASSO) Cox regression analysis were employed to construct the risk signature model. Receiver operation characteristic (ROC) curves were used to verify the prognostic and diagnostic efficiency. Immune infiltration level was evaluated by ESTIMATE algorithm and immune-related single-sample gene set enrichment analysis (ssGSEA).

**Results:**

High expression of HNRNPC, HNRNPA2B1, and YTHDF2 and low expression of ZC3H13 were associated with poor survival. Based on these four genes, a prognostic risk signature model was established. Multivariate Cox regression analysis demonstrated that the risk score was an independent prognostic factor of MM. Enrichment analysis showed that cell cycle, immune response, MYC, proteasome, and unfold protein reaction were enriched in high-risk MM patients. Furthermore, patients with higher risk score exhibited lower immune scores and lower immune infiltration level.

**Conclusion:**

The m6A-based prognostic risk score accurately and robustly predicts the survival of MM patients and is associated with the immune infiltration level, which complements current prediction models and enhances our cognition of immune infiltration.

## Introduction

Multiple myeloma (MM) is a neoplastic hematological malignancy characterized by clonal proliferation of malignant plasm cells in bone marrow (BM). MM has been the second most frequent hematological malignancy and accounts for 1.79% of all new cancer cases and 2.11% of all cancer deaths worldwide (1). The treatment arsenal in the battle of MM began with chemotherapy, and was rediscovered with proteasome inhibitor (PI)-based triple-drug combination, and extended with the splendid results achieved by immunotherapy, such as monoclonal antibodies (mAbs) and chimeric antigen receptor-engineered T cells (CAR-T). The survival of MM patients has dramatically prolonged in the last decade. Nonetheless, the medical needs of MM patients remain unmet, especially high-risk patients (2). Two leading obstacles for MM treatment are high recurrence rate and highly heterogeneous cell population whose subclone content evolves over time. Not only are genotypes and phenotypes of MM responsible for the high tumor heterogeneity but also the tumor microenvironment is implicated in it. Similar to what is observed in chemotherapy, MM presents capability to escape from immunotherapy, which is associated with high genetic instability and the protection of bone marrow microenvironment (BMME) corrupted by MM cells (3). Considerable research emphasizing the role of BMME in hematological cancers explicitly demonstrated that cellular components of bone marrow, including immune cell and stromal cell, can support the proliferation of MM cells and shield MM cells from the attack of chemotherapy and immune system (4). The alteration of immune infiltration level in BMME involves the development and recruitment of multiple immunosuppressive cells, such as regulatory T cells (Treg), myeloid-derived suppressor cells (MDSCs) and tumor-associated macrophages (TAMs) (5). In addition, the dysregulation of immune checkpoint genes, such as programmed cell death 1 ligand1/L2 and TIGIT, and the loss or downregulation of human leukocyte antigen (HLA) are also involved in the immune-suppressive status of BMME (6–8).

N6-methyladenosine (m6A) was first discovered in eukaryotic mRNA in the 1970s, and it has been recognized as the most abundant RNA modifications among more than 160 types of distinct chemically posttranscriptional modifications (9–11). However, it was not until 2012 that the first whole transcriptome high-throughput sequencing of m6A modification was finished, initiating the detailed investigation of m6A in various diseases, especially in cancers (12, 13). Similar to DNA and histone methylation, m6A RNA modification is dynamically reversible with the involvement of m6A “writers,” “erasers,” and “readers”. The installation of m6A is catalyzed by writers/methyltransferase complex (MTX), including methyltransferase-like protein 3 (METTL3), METTL14, METTL16, WT1-associated protein (WTAP), RNA-binding motif protein 15 (RBM15), RBM15B, KIAA1429 (or named VIRMA), and zinc finger CCCH-type containing 13 (ZC3H13). Eraser is a class of demethylases, including fat mass and obesity-associated protein (FTO), α-ketoglutarate-dependent dioxygenase homolog 5 (ALKBH5), and ALKBH3 (14). Read proteins can recognize specific m6A sites and regulate the translation, degradation, and splicing of target RNA. YT521-B homology domain family proteins 1/2/3 (YTHDF1/2/3) and YTH domain containing 1/2 (YTHDC1/2) are associated with RNA translation efficiency and degradation (15, 16). Heterogeneous nuclear ribonucleoprotein A2B1 (HNRNPA2B1) plays an important role in microRNA maturation. HNRNPC and HNRNPG, which can mediate RNA abundance and splicing, are considered as “indirect” readers upon their tendency to preferentially bind to an RNA structure switch induced by m6A modifications (17, 18). Insulin-like growth factor 2 mRNA-binding proteins 1/2/3 (IGF2BP1/2/3) can promote mRNA stability and translation. Emerging studies have demonstrated that aberrant m6A modification is involved in oncogenesis, metastasis, drug resistance, and antitumor immunity (19–22). Dali et al. (22) observed that YTHDF1-deficient mice presented an enhanced CD8+ T cell infiltration level and the improved therapeutic efficacy of PD-L1 checkpoint inhibitor. However, in MM, very few studies also have revealed the involvement of m6A-related genes in oncogenesis and progression at single gene level (23–26). Research focusing on the systematic cognition of the role of m6A in the prognosis and immune microenvironment of MM is warranted.

In the present study, we used the gene expression data and clinical information from the Gene Expression Omnibus (GEO) database to explore differentially expressed and prognosis-related m6A regulators. Then, we established a four-gene prognostic risk signature model using least absolute shrinkage and selection operator (LASSO) Cox regression analysis to evaluate the survival outcomes of MM patients. Finally, enrichment analysis and the evaluation of immune infiltration level were performed. Our study systematically dissected the abnormal expression status of m6A-related genes and revealed the role of m6A RNA methylation modification in the prognosis and immune infiltration of MM, which complements current prediction models and enhances our cognition of immune infiltration.

## Materials and Methods

### Data Preparation

The gene expression matrix and clinical information of all samples were downloaded from the NCBI GEO database (https://www.ncbi.nlm.nih.gov/geo/). The raw count data was preprocessed with normalization and log2 transformation. The overall design and workflow of this study is given in **Supplementary Figure S1**. Briefly, The GSE47552 was applied for the identification of differentially expressed m6A-related genes. The GSE47552, GSE6477, and GSE13591 were used for the evaluation of risk score for MM diagnosis. Three independent series GSE24080, GSE9782, and GSE57317 were identified using the following selection criteria: (1) >40 subjects, (2) available survival data, and (3) confirmed MM patients. Three hundred thirteen MM patients from the GSE24080 was used as the training cohort for model construction, and the GSE9782 and GSE57319 were used as validation cohorts. Detailed information of all datasets used in this study are shown in **Supplementary Table S1**.

### Identification of Differentially Expressed m6A RNA Methylation Regulators

We systematically analyzed the expression of 22 m6A-related genes according to previously published articles (27), including 7 writers (METTL3, METTL14, WTAP, KIAA1429, RBM15, RBM15B, and ZC3H13), 3 erasers (FTO, ALKBH5, and ALKBH3), and 12 readers (IGF2BP1/2/3, YTHDF1/2/3, YTHDC1/2, HNRNPA2B1, HNRNPC, and RBMX) in GSE47552. The heatmap was generated *via* the PHEATMAP package (version 1.0.12; https://cran.rstudio.com/web/packages/pheatmap/index.html).

### Protein–Protein Interaction Network and Spearman Correlation Analysis

The PPI network among the differentially expressed m6A regulatory genes was constructed using the STRING online database (www.string-db.org). Cytoscape software (version 3.8.2) was used for visualization (28). Spearman correlation analysis was performed to demonstrate the association among different m6A-related genes based on the Spearman’s correlation coefficient (*r*) value between −1 and +1. The CORRPLOT package (version 0.84; https://github.com/taiyun/corrplot) was used to visualize the correlation matrix.

### Construction of the Prognostic Risk Model

First, all MM patients in the training cohort were divided into two groups according to the mean expression level of each gene. Kaplan–Meier survival analysis with log-rank test was used to evaluate the survival rate by the “SURVIVAL” package (version 3.2-7; https://github.com/therneau/survival). We selected four genes (ZC3H13, HNRNPA2B1, HNRNPC, and YTHDF2) showing significant correlation with the overall survival (OS) as prognostic-related genes. Second, we performed the least absolute shrinkage and selection operator (LASSO) Cox regression analysis to select the optimal weighting coefficient and build the risk model with the “glmnet” package (version 4.1-1; https://glmnet.stanford.edu/). Optimal values of penalty parameter lambda were determined by 1,000-fold cross-validation *via* the minimum criteria (29). The risk score formula was as follows: risk score = (−0.4820058*expression value of ZC3H13) + (0.4641464*expression value of HNRNPA2B1) + (0.3691959*expression value of HNRNPC) + (0.2454545*expression value of YTHDF2). Third, we divided MM patients of the training cohort into high- and low-risk groups upon the optimal cutoff of the risk score with the “Survminer” package (version 0.4.9; https://rpkgs.datanovia.com/survminer/index.html) and analyzed the survival rate by Kaplan–Meier survival analysis. Subsequently, the predictive performance of the m6A risk score was assessed using ROC curves and the value of area under the ROC curve (AUC). The reliability and stability of this prognostic risk model was further ensured in two validation cohorts.

### Establishment and Assessment of the Nomogram

Univariate and multivariate Cox regression analyses were employed to evaluate the prognostic efficiency of other clinical features and to identify whether the risk score was an independent prognostic factor. Briefly, variables with *p* < 0.10 in univariate analysis were eligible for multivariate Cox regression analysis with forward likelihood ration (LR) method, and *p* < 0.05 was considered statistically different. Then, the nomogram with independent prognostic factors was constructed using the “rms” package (version 6.2-0; https://hbiostat.org/R/rms/). The predictive performance of the nomogram was evaluated with calibration curves and ROC curves.

### Differential Expression Analysis

Three hundred thirteen MM patients from the GSE24080 were stratified into high- and low-risk groups according to the cutoff value in the survival analysis. Differential expression analysis was applied using the LIMMA package (30) (version 3.46.0; http://bioinf.wehi.edu.au/limma/). An absolute log2 fold change (FC) > 0.6 and adjusted *p* < 0.05 was used as the cutoff for differentially expressed genes (DEGs).

### Enrichment Analysis

The online website WebGestalt (http://www.webgestalt.org/) (31) was applied to analyze enriched biological terms of DEGs, including Gene Ontology (GO) and Kyoto Encyclopedia of Genes and Genomes (KEGG) pathway. Gene set enrichment analysis (GSEA) was conducted with GSEA software (version 4.1.0). Official gene sets, including Hallmark (H), KEGG (C2), and Gene ontology (C5), were downloaded from GSEA website (http://www.gsea-msigdb.org/gsea/msigdb/index.jsp) for enrichment. A permutation number of 1,000 was adopted. A *p*-value cutoff of 0.05 with a false discovery rate (FDR *q*-value) < 0.05 was considered statistically significant.

### Evaluation of Immune infiltration Level

The ESTIMATE algorithm (32) was used to calculate the immune score, stromal score, and tumor purity of each sample for preliminary evaluation. Next, we obtained gene sets marking each tumor microenvironment infiltration immune cell type from previous studies (33, 34). We performed single sample GSEA (ssGSEA) to derive the enrichment score of each immune-associated gene set to represent the relative abundance of each infiltrating cell in each sample by using “GSVA” package (version 1.38.2; https://github.com/rcastelo/GSVA).

### Statistical Analysis

Statistical analysis was conducted using SPSS 26.0, R Studio (version 1.4.1103; https://rstudio.com/) and GraphPad Prism software (version 8.0.2). Continuous variables were described as the mean ± standard deviation (SD); Mann–Whitney test and Student’s *t*-test were used to compare the difference in subgroups as appropriate. One-way ANOVA followed by Bonferroni *post-hoc* comparison were employed to compare the difference of more than two subgroups. *p* < 0.05 was considered statistically significant if not specified. All statistical tests were two-sided.

## Results

### Nine of 22 m6A-Related Genes Were Differentially Expressed in MM

**Figure 1:**
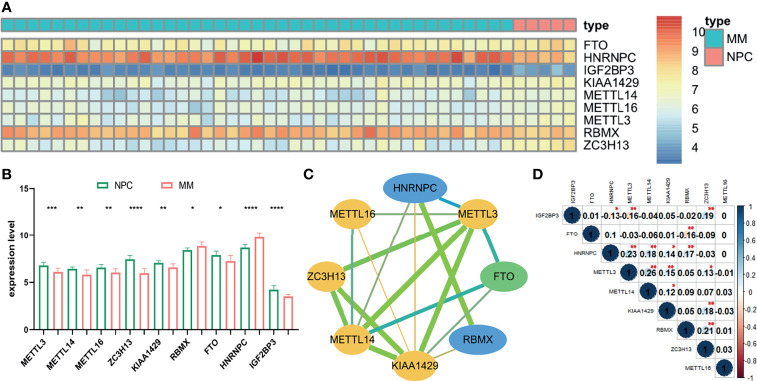
The expression status and correlation of differentially expressed m6A-related genes in MM. The heatmap **(A)** and the bar chart **(B)** showed the expression of nine differentially expressed m6A-related regulators in 41 MM and 5 NPC samples from the GSE47552 dataset. The color bar of heatmap from red to blue denotes high to low gene expression. **(C)** The PPI network of the differentially expressed m6A-related genes. Yellow, green, and blue nodes represent “writer,” “eraser,” and “reader,” respectively. Node size represents the expression value. Edge size represents the combined score (low score to orange and high score to green). **(D)** The Spearman correlation analysis of the differentially expressed m6A-related genes. The size of each circle denotes the Spearman correlation coefficient (negativity to red and positivity to blue). *p < 0.05, **p < 0.01, ***p < 0.001, ****p < 0.0001.

The gene expression of 22 m6A-related genes in 5 normal plasm cell (NPC) samples and 41 MM patients from the GSE47552 was used for differentially expressed analysis. The heatmap showed that the expression of nine m6A methylation regulators (FTO, HNRNPC, IGF2BP3, KIAA1429, METTL16, METTL14, METTL3, RBMX, and ZC3H13) were differentially expressed in MM patients compared to normal volunteers (**Figure 1A**). We observed that RBMX and HNRNPC were significantly upregulated, while METTL3, METTL14, METTL16, ZC3H13, KIAA1429, FTO, and IGF2BP3 were significantly downregulated in MM patients (**Figure 1B**). Furthermore, we used the online database cBioPortal (http://www.cbioportal.org/) to determine the mutant frequency of m6A-related genes in MM samples; however, no frequent mutation was found (**Supplementary Figure S2**) (35, 36).

### PPI Network and Spearman Correlation Analysis Among m6A RNA Methylation Regulators

To further investigate the relationship among nine m6A RNA methylation regulators, PPI network was established (**Figure 1C**), which demonstrated that KIAA1429 and METTL3 were hub genes. In addition, IGF2BP3 had no protein–protein association with others, which was hidden in the figure. Moreover, the correlation of each gene expression level was analyzed by the Spearman correlation analysis (**Figure 1D**). The correlation between METTL3 and METTL14 was most positively significant (*r* = 0.26), and the relationship between METTL3 and IGF2BP3, FTO, and RBMX were most significantly negative (*r* = −0.16).

### Construction and Validation of the Prognostic Risk Model Based on Four m6A Methylation Regulators

**Figure 2:**
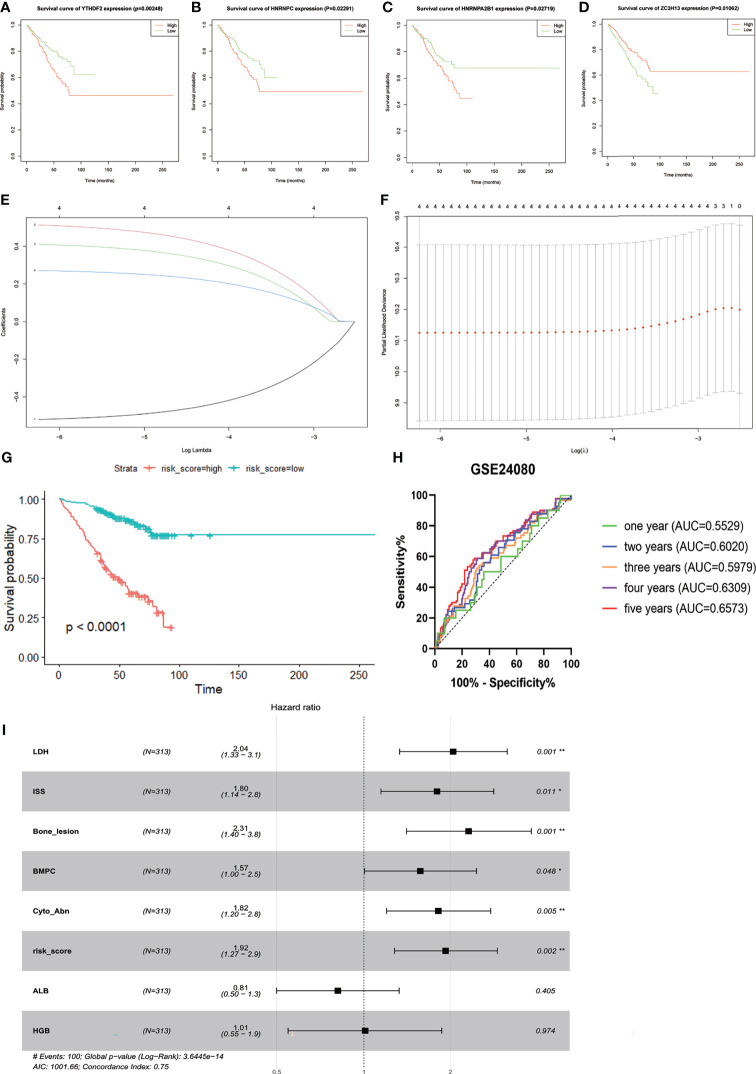
Construction and evaluation of the four-gene prognostic risk signature in the training cohort of MM patients. Kaplan–Meier survival curves for YTHDF2 **(A)**, HNRNPC **(B)**, HNRNPA2B1 **(C)**, and ZC3H13 **(D)**. **(E)** The LASSO Cox analysis identified four m6A-related genes in the training cohort. Each curve corresponds to one gene (red, HNRNPA2B1; green, HNRNPC; blue, YTHDF2; black, ZC3H13). **(F)** Partial likelihood deviance of different numbers of variables. One-thousand-fold cross-validation was applied for tuning penalty parameter selection. **(G)** Kaplan–Meier survival analysis by different risk score levels in training cohort. **(H)** ROC curve was applied to assess the predictive efficiency of the prognostic risk signature. **(I)** Forest plot of the multivariate Cox regression analysis. *P<0.05, **P<0.01.

Three hundred thirteen IgG MM patients from the GSE24080 were divided into low- and high-expression groups according to the mean expression level of each m6A-related gene. We found that the overexpression of YTHDF2 (*p* = 0.00248), HNRNPC (*p* = 0.02291), and HNRNPA2B1 *(p* = 0.02719) was associated with the poor OS, and survival outcomes of patients with high ZC3H13 expression were significantly prolonged than that of patients with low expression (*p* = 0.01062, **Figures 2A–D**). However, the expression levels of other genes were not correlated with OS (**Supplementary Figure S3**). Subsequently, based on the result of LASSO Cox regression analysis using the penalized maximum likelihood estimator and min criteria for the optimal lambda value, four m6A methylation regulators, including ZC3H13, HNRNPA2B1, HNRNPC, and YTHDF2, were identified to construct the risk model (**Figures 2E, F**). Then, we calculated the risk score for each patient of the training cohort and stratified them into low- and high-risk groups according to the optimal cutoff value (7.060, calculated by R program). Kaplan–Meier survival analysis showed that MM patients in high-risk group had significantly shorter OS than patients in low-risk group (*p* < 0.0001, **Figure 2G**). The ROC curve analysis was used to investigate the predictive specificity and sensitivity of the risk score. The AUC values of the risk score for 1-, 2-, 3-, 4-, and 5-year survival were 0.5529 (95%CI, 0.4202–0.6856, *p* = 0.4286), 0.6020 (95%CI, 0.5089–0.6952, *p* = 0.0351), 0.5979 (95%CI, 0.5170–0.6789, *p* = 0.0185), 0.6390 (95%CI, 0.5646–0.7133, *p* = 0.0004), and 0.6573 (95%CI, 0.5785–0.7360, *p* = 0.0002), respectively (**Figure 2H**).The AUC values indicated that the risk score had a great discrimination ability for the prognosis of MM patients.

**Figure 3:**
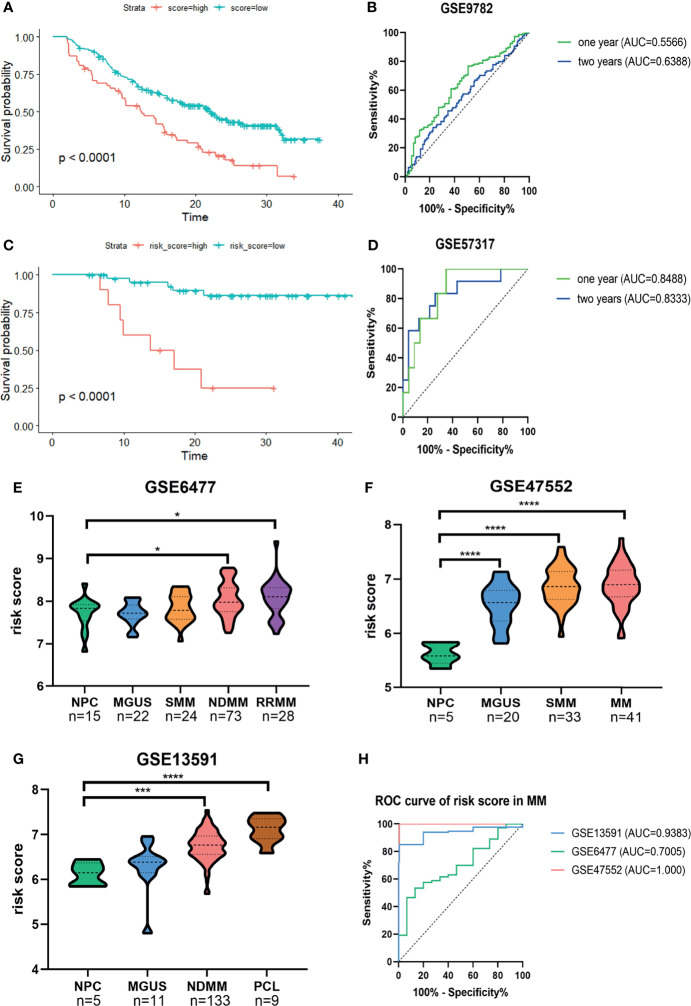
Confirmation of the predictive performance of risk signature for MM prognosis and diagnosis in validation cohorts. **(A, B)** Kaplan-Meier survival analysis and ROC curve in the GSE9782 dataset. **(C, D)** Kaplan-Meier survival analysis and ROC curve in GSE57317 dataset. **(E–G)** The risk score levels of the continuous disease spectrum in MM datasets GSE6477 **(E)**, GSE47552 **(F)** and GSE13691 **(G)**. **(H)** ROC curves of risk score in MM to assess the diagnostic efficiency. *P<0.05, ***P<0.001, ****P<0.0001.

Next, Kaplan–Meier survival analysis and ROC curve for the risk score were conducted in two validation cohorts. The OS of low-risk patients was significantly longer than that of high-risk patients in the GSE9782 (*p* < 0.0001, **Figure 3A**) and GSE57317 (*p* < 0.0001, **Figure 3C**). The AUC values of the risk score for 1- and 2-year survival were 0.5566 (95%CI, 0.4827–0.6305, *p* = 0.1329) and 0.6388 (95%CI, 0.5558–0.7219, *p* = 0.0019) in the GSE9782 (**Figure 3B**). Parallel values in the GSE57137 were 0.8488 (95%CI, 0.7224–0.9753, *p* = 0.0061) and 0.8333 (95%CI, 0.6841–0.9825, *p* = 0.0014, **Figure 3D**). Our results demonstrated the great efficiency of the prognostic risk score for MM patients.

The development of MM has been widely identified as a multistage process starting with the premalignant condition monoclonal gammopathy of unknown significance (MGUS), followed by the intermediate stage smolder MM (SMM), and some progressed into the refractory and/or relapse MM (RRMM) or plasma cell leukemia (PCL). We observed that the risk score was significantly increased with the development of MM (**Figures 3E–G**). The ROC curves showed that the risk score could discriminate between MM and normal individuals with high sensitivity and specificity; the AUC values were 0.9383 (95%CI, 0.8854–0.9913, *p* = 0.0009), 0.7005 (95%CI, 0.5703–0.8306, *p* = 0.0149), and 1.000 (95%CI, 1.000–1.000, *p* = 0.0003) in GSE13591, GSE6477, and GSE47552, respectively (**Figure 3H**).

### The Risk Score as an Independent Prognostic Factor

**Figure 4:**
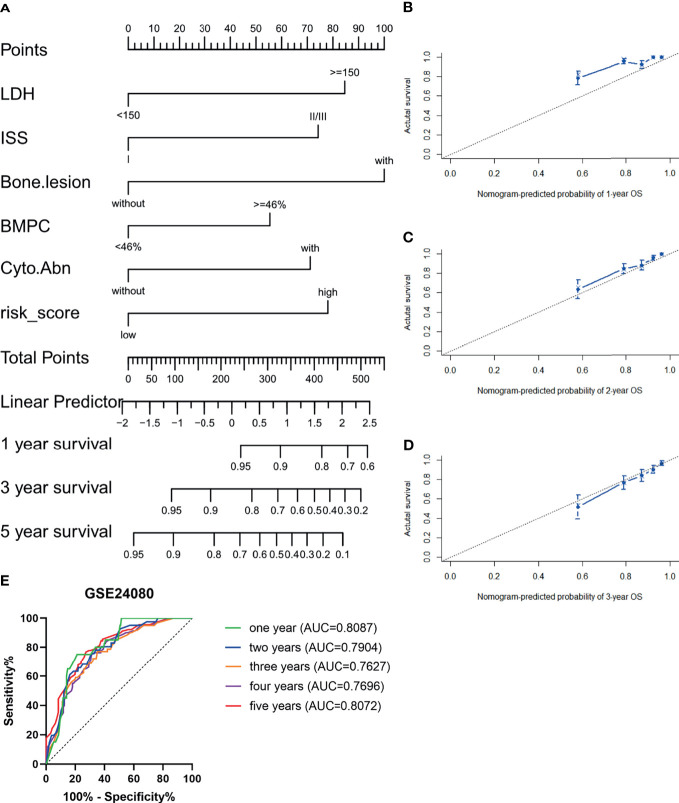
Establishment and assessment of the nomogram. **(A)** Nomogram predicting 1-, 3-, and 5-year survival for MM patients in the training cohort based on the risk score and other clinical parameters. **(B–D)** Calibration plot of the nomogram for 1-year **(B)**, 2-year **(C)**, and 3-year **(D)** OS in the training cohort. **(E)** ROC curve in the GSE24080 for the nomogram risk score system.

The GSE24080 was used for univariate and multivariate Cox regression analyses to identify the independent prognostic factor of the risk score and other relevant clinicopathological factors, including age, gender, race, lactic dehydrogenase (LDH), albumin (ALB), hemoglobin (HGB), ISS stage, bone marrow plasm cell (BMPC), bone lesion, and cytogenetic abnormality. The results showed that LDH [hazard ratio (HR), 2.040; 95%CI, 1.327–3.137; *p* = 0.001], ISS stage (HR, 1.869; 95%CI, 1.217–2.869; *p* = 0.004), BMPC (HR, 1.601; 95%CI, 1.027–2.497; *p* = 0.038), cytogenetic abnormalities (HR, 1.819; 95%CI, 1.202–2.753, *p* = 0.005), bone lesion (HR, 2.323; 95%CI, 1.411–3.826; *p* = 0.001), and the risk score (HR, 1.928; 95%CI, 1.278–2.907; *p* = 0.002) were significantly associated with OS (**Table 1** and **Figure 2I**), which indicated that the risk score based on four m6A regulators is an independent prognostic factor for MM patients. To quantify the OS predication, we integrated the risk sore and independent clinical features (LDH, ISS stage, BMPC, bone lesion, and cytogenetic abnormalities) to construct a nomogram (**Figure 4A**). The calibration curves illustrated a great agreement between predicted and actual survival probabilities (**Figures 4B–D**). The nomogram risk score system greatly improved the prognostic risk signature model. The AUC values of the nomogram risk score for 1-, 2-, 3-, 4-, and 5- year survival were 0.8087 (95%CI, 0.7327–0.8847; *p* < 0.0001), 0.7904 (95%CI, 0.7242–0.8566; *p* < 0.0001), 0.7627 (95%CI, 0.6991–0.8262; *p* < 0.0001), 0.7696 (95%CI, 0.7091–0.8301; *p* < 0.0001), and 0.8072 (95%CI, 0.7457–0.8688; *p* < 0.0001), respectively (**Figure 4E**).

**Table 1:** Univariate and multivariate analyses of OS using the Cox proportional hazard regression model.

Variables	Univariate analysis		Multivariate analysis				
	HR (95%CI)	*p*-value	Beta	SE	Wald	HR (95%CI)	*p*-value
Age, years	0.953 (0.593–1.531)	0.842					
Gender	1.103 (0.733–1.660)	0.638					
Race	1.145 (0.639–2.052)	0.649					
LDH (U/L)	2.564 (1.685–3.903)	**<0.0001**	0.713	0.22	10.548	2.040 (1.327–3.137)	**0.001**
ALB (g/L)	0.533 (0.339–0.838)	**0.006**	−0.2073	0.2487	−0.83	0.812 (0.499–1.323)	0.403
HGB (g/dL)	0.546 (0.310–0.961)	**0.036**	0.01	0.3133	0.03	1.015 (0.549–1.875)	0.963
ISS stage	2.531 (1.705–3.759)	**<0.0001**	0.625	0.219	8.162	1.869 (1.217–2.869)	**0.004**
BMPC	2.118 (1.404–3.197)	**<0.0001**	0.471	0.227	4.318	1.601 (1.027–2.497)	**0.038**
Cytogenetic abnormalities	2.662 (1.788–3.961)	**<0.0001**	0.598	0.211	8.016	1.819 (1.202–2.753)	**0.005**
Bone lesions	2.246 (1.374–3.670)	**0.001**	0.843	0.255	10.97	2.323 (1.411–3.826)	**0.001**
Risk score	2.381 (1.599–3.546)	**<0.0001**	0.656	0.21	9.807	1.928 (1.278–2.907)	**0.002**

The bold values indicate statistically different (P<0.05).

### Subgroup Analysis

**Figure 5:**
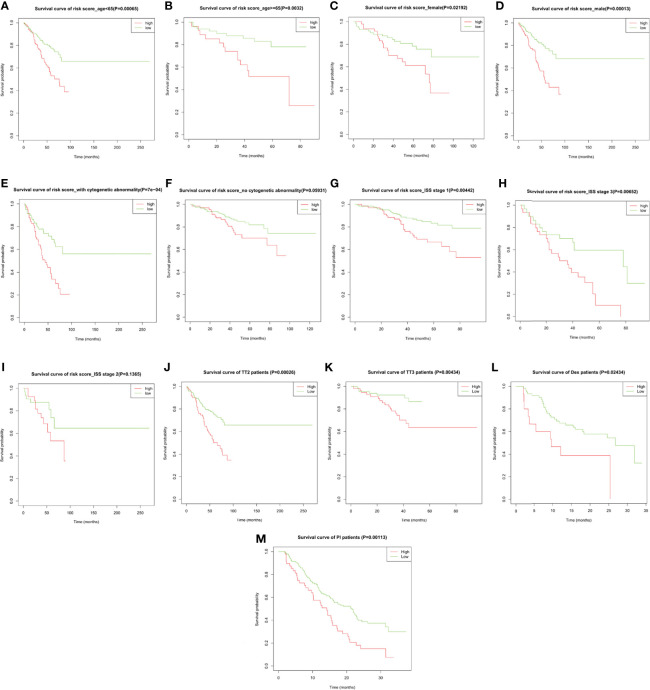
The overall survival probability in high- and low-risk MM patients grouped by clinicopathological features and therapeutic regimens. Kaplan–Meier survival analysis of MM patients from GSE24080 stratified by **(A, B)** age ≤65 and >65, **(C, D)** female and male, **(E, F)** with and without cytogenetic abnormalities, **(G–I)** ISS stage I/II/III, and **(J, K)** total therapy 2 (TT2) and TT3 regimen. Kaplan–Meier survival analysis of MM patients from GSE9782 stratified by **(L, M)** dexamethasone (DEX) treatment and proteasome inhibitor (PI) treatment.

Subgroup analysis upon age, gender, International Staging System (ISS), cytogenetic abnormality in the training cohort, and upon therapeutic strategies in both the training cohort and the validation cohort GSE9782 was performed to evaluate the accuracy and stability of the risk score for different patients. The risk score presented stable predictive efficiency in all age and gender (*p* < 0.05, **Figures 5A–D**). Furthermore, higher risk score was correlated with poorer OS in patients with cytogenetic abnormalities (*p* = 7e−04, **Figure 5E**), while no significant survival difference was observed in patients without cytogenetic abnormalities (*p* = 0.05931, **Figure 5F**). ISS I- and ISS III-stage MM patients with high risk score had significantly shorted OS than those with low risk score (*p* = 0.00442 and 0.00652, respectively; **Figures 5G, H**), while no significant difference was observed in ISS II stage (*p* = 0.1365; **Figure 5I**). In addition, subgroup analysis for therapeutic regimens verified the robustness of the prognostic risk signature in different treatment choices, including total therapy 2 (TT2, a combination treatment with thalidomide, *p* = 0.00026, **Figure 5J**), TT3 (a combination treatment with the addition of bortezomib, *p* = 0.00434, **Figure 5K**), dexamethasone (DEX, *p* = 0.02434, **Figure 5L**), and PS341 (a kind of proteasome inhibitor, *p* = 0.00113, **Figure 5M**), which demonstrated that the prognostic risk score meets individualized therapeutic needs of MM patients.

### Correlation Between the Prognostic Risk Score and Clinical Characteristics

We then analyzed the correlation between the risk score and the clinicopathological features of MM patients in the GSE24080 cohort (**Table 2**). We observed significantly increased risk scores regarding advanced ISS stage (*p* = 0.013). Moreover, MM patients with cytogenetic abnormalities were observed to have higher risk scores than those cytogenetically normal (*p* < 0.0001). Subsequently, we analyzed the relationship between the expression of the four prognostic risk signature genes and clinical features (**Supplementary Table S2**). We observed that high expression of YTHDF2 and HNRNPA2B1 were associated with advanced ISS stage. The higher expression of HNRNPA2B1 was correlated with higher BMPC ratio. Additionally, MM patients with cytogenetic abnormalities were observed to have higher HNRNPC expression and lower ZC3H13 expression.

**Table 2:** The correlation of prognostic risk signature and clinical characteristics.

Variables	Number	Risk score, mean(SD)		*p-*value
Age, years				0.364
<65	236	7.0106 (0.36658)		
≥65	77	6.9681 (0.32058)		
Gender				0.967
Female	118	7.0012 (0.34289)		
Male	195	6.9995 (0.36424)		
Race				0.419
White	270	6.9936 (0.35399)		
Others	43	7.0409 (0.36849)		
LDH (U/L)				0.755
<150	156	6.9938 (0.31829)		
≥150	157	7.0064 (0.39043)		
ALB (g/L)				0.086
<3.5	57	7.0732 (0.33478)		
≥3.5	256	6.9838 (0.35890)		
HGB (g/dl)				0.133
<9.0	26	7.1005 (0.34428)		
≥9.0	287	6.9910 (0.35600)		
ISS stage				**0.013**
I	194	6.9602 (0.34356)		
II	59	7.0173 (0.39841)		
III	60	7.1123 (0.33001)		
BMPC				0.209
<46%	157	6.9749 (0.33608)		
≥46%	156	7.0255 (0.37394)		
Cytogenetic abnormalities				**<0.0001**
Yes	117	7.0913 (0.35062)		
No	196	6.9456 (0.34847)		
Bone lesions				0.616
Yes	210	7.0072 (0.37301)		
No	103	6.9857 (0.31907)		

The bold values indicate statistically different (P<0.05).

### Differential Expression Analysis and Functional Enrichment Analysis

**Figure 6:**
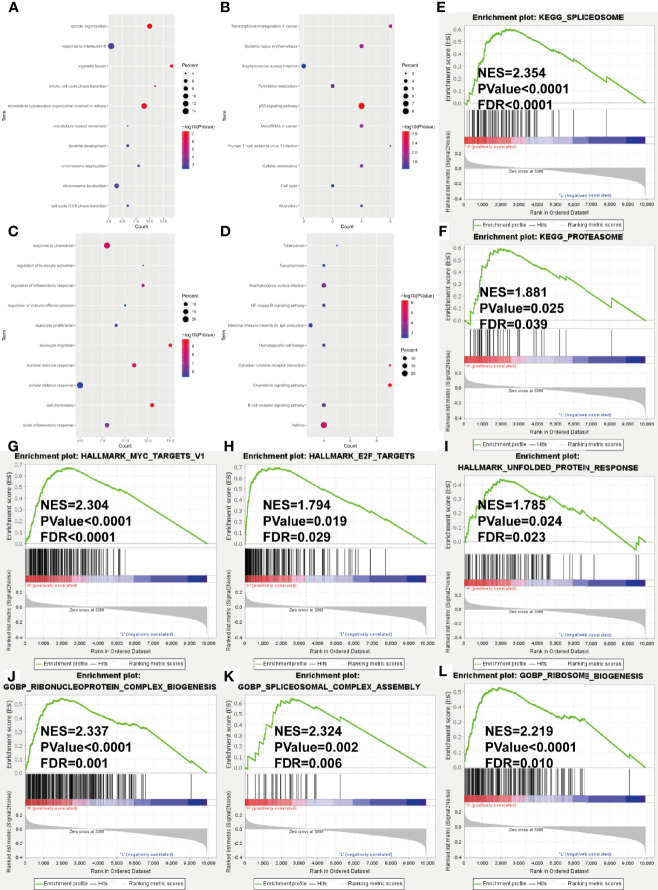
Gene ontology and KEGG pathway enrichment analysis of DEGs in GSE24080 dataset grouped by risk score level. **(A–D)** Bubble diagrams show the significantly enriched gene ontology biological process (GOBP) and KEGG pathway terms of upregulated DEGs **(A, B)** and downregulated DEGs **(C, D)**. **(E–L)** GSEA results of KEGG gene set **(E, F)**, HALLMARK gene set **(G–I)** and GOBP gene set **(J–L)**. NES, normalized enrichment score; FDR, false discovery rate.

In the training cohort, we conducted the differential expression analysis between high- and low-risk groups. A total of 156 upregulated and 90 downregulated genes were identified to be significantly associated with the risk score (**Supplementary Table S3** and **Supplementary Figure S4**). Then, the functional enrichment analysis observed that immune-related biological processes and malignant hallmarks were enriched (**Figures 5A–D**). The upregulated genes showed significantly enriched GO terms, including response to interleukin-6, mitotic cell cycle phase transitions, and KEGG terms, including transcriptional misregulation in cancer, p53 signaling pathway, and cell cycle (**Figures 6A, B**). GO terms, including response to chemokine, regulation of inflammatory response, and leukocyte proliferation, and KEGG terms, including nuclear factor (NF)-kappa B signaling pathway, hematopoietic cell lineage, and cytokine–cytokine receptor interaction, were significantly enriched in downregulated genes (**Figures 6C, D**). Furthermore, GSEA found significantly enriched terms related to tumorigenesis and drug resistance in high-risk group (**Supplementary Table S4**), including spliceosome (NES = 2.354, *p* < 0.0001, FDR < 0.0001, **Figure 6E**) and proteasome (NES = 1.881, *p* = 0.025, FDR = 0.039, **Figure 6F**) in KEGG gene set; MYC targets (NES = 2.304, *p* < 0.0001, FDR < 0.0001, **Figure 6G**), E2F targets (NES = 1.794, *p* = 0.019, FDR = 0.029, **Figure 6H**), and unfold protein response (NES = 1.785, *p* = 0.024, FDR = 0.023, **Figure 6I**) in HALLMARK gene set; ribonucleoprotein complex biogenesis (NES = 2.337, *p* < 0.0001, FDR = 0.001, **Figure 6J**), spliceosomal complex assembly (NES = 2.324, *p* = 0.002, FDR = 0.006, **Figure 6K**), and ribosome biogenesis (NES = 2.219, *p* < 0.0001, FDR = 0.010, **Figure 6L**) in GOBP gene set.

### Association of the Risk Score With the Immune Microenvironment

**Figure 7:**
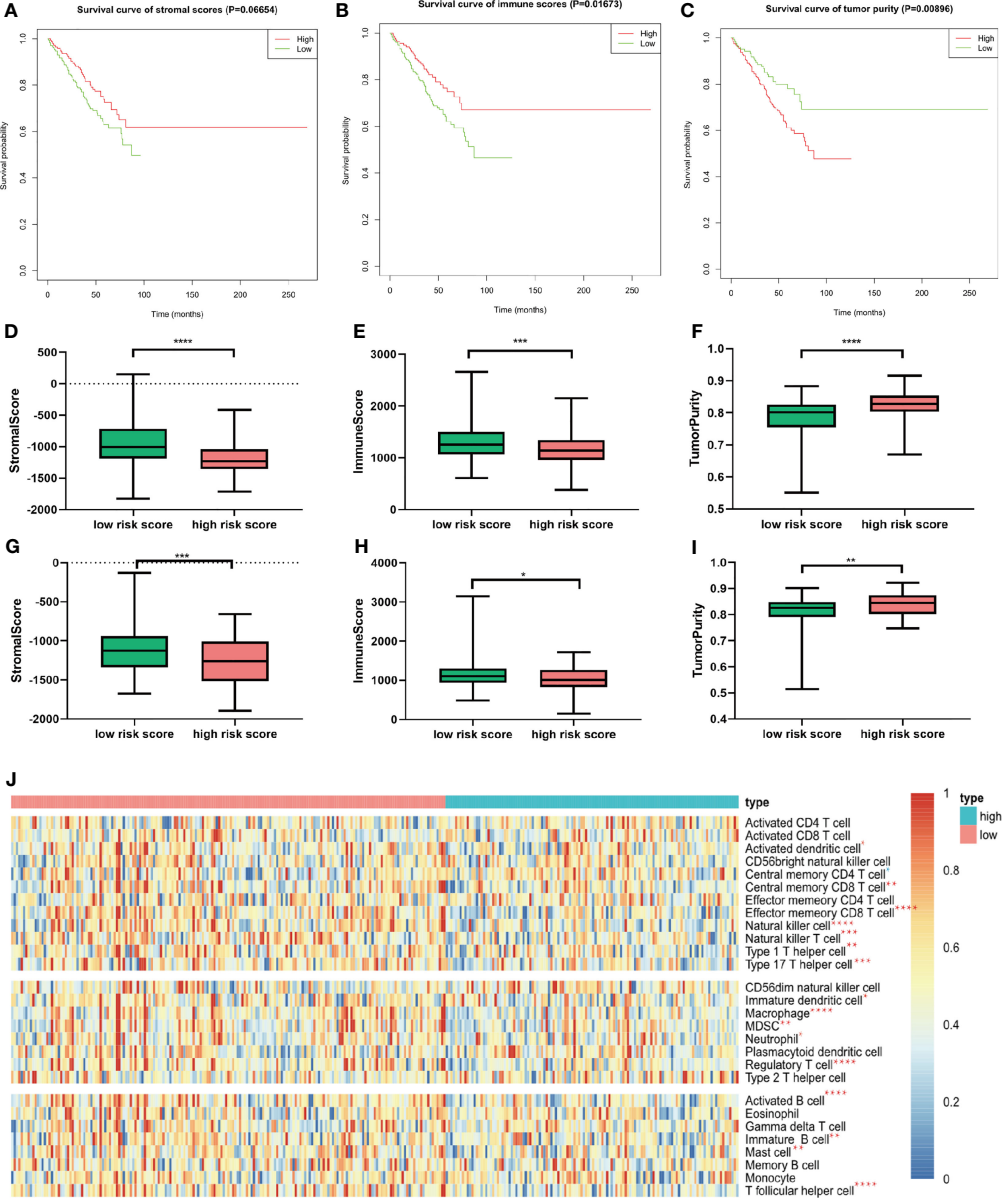
Relationship between immune infiltration level and m6A prognostic risk score. **(A–C)** Kaplan–Meier survival analysis upon stromal score **(A)**, immune score **(B)**, and tumor purity **(C)** in GSE24080. **(D–F)** The distribution of stromal score **(D)**, immune score **(E)**, and tumor purity **(F)** upon different risk score in GSE24080. **(G–I)** The distribution of stromal score **(D)**, immune score **(E)**, and tumor purity **(F)** upon different risk score of the validation cohort GSE9782. **(J)** Twenty-eight immune-related gene sets were performed for ssGSEA. Seventeen immune cell gene sets were significantly different between patients with high and low risk score. Red star represents higher enrichment score; blue star represents lower enrichment score compared to high-risk MM patients. *p < 0.05, **p < 0.01, ***p < 0.001, ****p < 0.0001.

Given that considerable immune-related terms were substantially enriched in DEGs based on the risk score, we speculated that the risk score was correlated with immune infiltration level. To verify our hypothesis, we assessed the immune infiltration level by the ESTIMATE algorithm. Then, MM patients were divided into high- and low-score groups according to the median value. Kaplan–Meier survival analysis revealed that MM patients with higher immune score and lower tumor purity had longer OS (*p* = 0.01673 and 0.00896, respectively, **Figures 7A–C**). Patients with high risk score in the training cohort (**Figures 7D–F**) and validation cohort (**Figures 7G–I**) had lower immune score and higher tumor purity, which indicated poorer prognosis. Furthermore, the implementation of ssGSEA was used for identifying the alteration of immune cell population upon the risk score. We observed that 16 of 28 immune cell subsets were significantly decreased in the high-risk group (**Figure 7J**). Antitumor immune cells, such as effector memory CD8 T cell, natural killer cell, natural killer T cell, type 1 T helper cell, and type 17 helper cell, were substantially reduced. However, immunosuppressive cells, including MDSC, regulatory T cell, and immature dendritic cell were also decreased in patients with high risk score.

**Figure 8:**
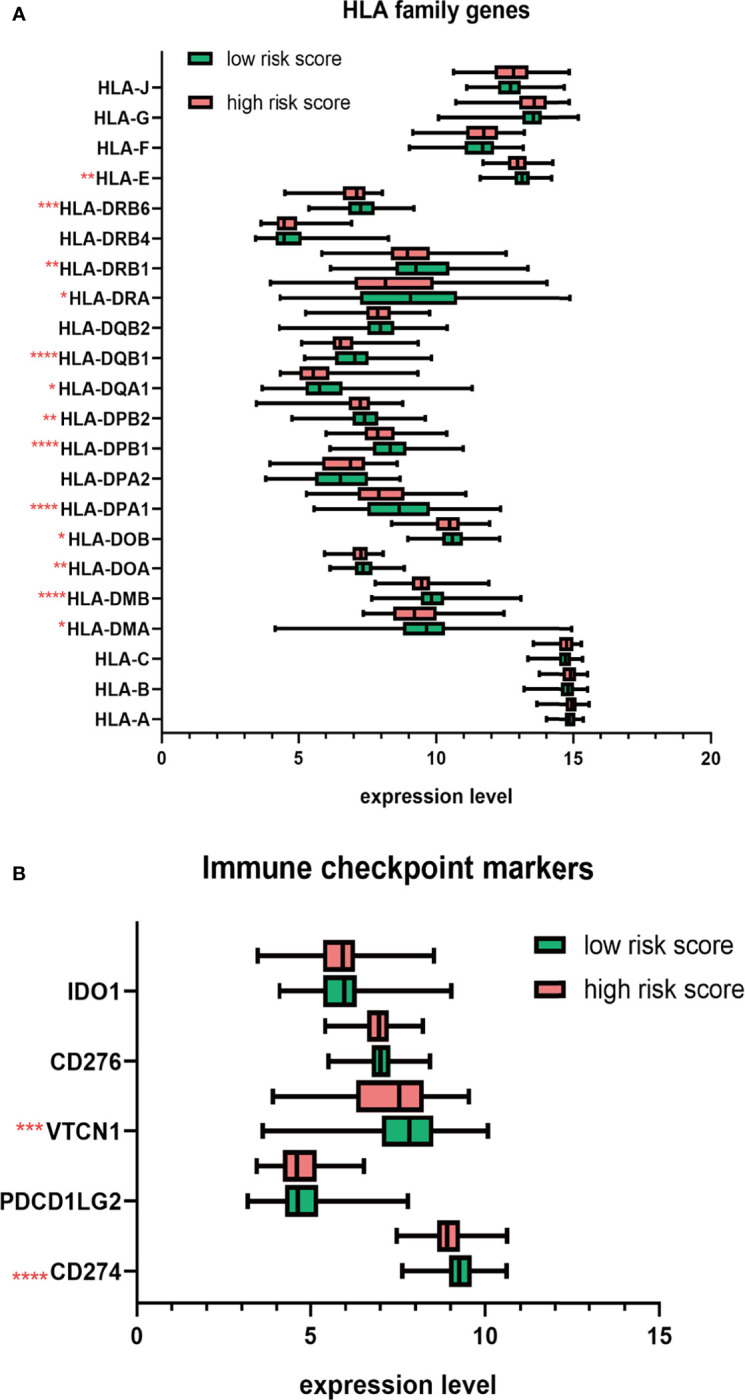
The distribution HLA family genes and immune checkpoint markers between the high- and low-risk MM patients. **(A)** Boxplot of HLA family genes. **(B)** Boxplot of immune checkpoint markers. *p < 0.05, **p < 0.01, ***p < 0.001, ****p < 0.0001.

Next, we studied the expression of HLA system and immune checkpoints, including CD274 (PD-L1), PDCD1LG2, VTCN1 (v-set domain-containing T cell activation inhibitor), CD276, and IDO1 (indoleamine 2,3-dioxygenase 1) (37), which were found on the surfaces of cells to help the recognition of immune system and involved in the immune activation and inhibition. We observed that the HLA family, including HLA-DMA, HLA-DMB, HLA-DOA, HLA-DOB, HLA-DPA1, HLA-DPB1, HLA-DPB2, HLA-DQA1, HLA-DQB1, HLD-DRA, HLA-DRB1, HLA-DRB6, and HLA-E, showed significant decrease in high-risk group (**Figure 8A**). Immune checkpoint markers, including CD274 and VTCN1, were significantly downregulated in patients with high risk score (**Figure 8B**).

## Discussion

N6-methyladenosine RNA modification, the most remarkable epitranscriptomic modification, has been widely considered to be involved in tumorigenesis and immune regulation, while its role in MM progression and bone marrow immune microenvironment is little known yet. In this study, we conducted the comprehensive bioinformatics analysis to explore the significance of m6A RNA methylation regulators in MM. We identified the aberrant expression status of m6A RNA methylation regulators in MM: RBMX and HNRNPC were significantly upregulated, while METTL3, METTL14, METTL16, ZC3H13, KIAA1429, FTO, and IGF2BP3 were significantly downregulated in MM patients compared to normal plasm samples. Furthermore, our results elucidated that the overexpression of HNRNPA2B1, HNRNPC, and YTHDF2, and the downregulation of ZC3H13 were negatively associated with the overall survival. Based on these four genes, a prognostic risk signature model was established *via* LASSO Cox regression analysis. Kaplan–Meier survival analysis showed that MM patients in the high-risk group had significantly shorter OS than patients in low-risk group. Two validation cohorts confirmed the predictive efficiency of the m6A risk score by ROC curve analysis. In addition, univariate and multivariate Cox regression analyses manifested that the risk score was an independent prognostic factor. To determine the clinical feasibility of the prognostic risk signature in MM, we assessed the correlation between the risk score and significant clinical characteristics. Subgroup survival analysis upon age, gender, ISS stage, cytogenetic abnormality, and different therapeutic strategies showed that the risk score was precise and robust for the individualized medical demands of MM patients. Additionally, we found that advanced ISS stage and the existence of cytogenetic abnormalities were positively associated with the risk score.

Consistent with published articles, HNRNPC can serve as an oncogene, which is overexpressed in various cancers. HNRNPC knockdown arrested the proliferation of breast cancer cell lines *via* activating the antitumor cascade of interferon response initiated by the cytoplasmic RNA sensors retinoic acid-inducible gene I (RIG-I) (38). ZC3H13 is involved in the assembly of m6A methyltransferase complex. In colorectal cancer, ZC3H13 acting as a tumor suppressor could suppress cancel cells proliferation and invasion *via* inactivating Ras-ERK signaling pathway (39). YTHDF2, as a member of m6A “reader,” can selectively recognize and bind to m6A-containing RNA to initiate its degradation. The overexpression of YTHDF2 in hepatocellular carcinoma (HCC) promoted stemness and metastasis *via* increasing OCT4 translation (40). In glioblastoma (GBM) stem cells, YTHDF2 was preferentially expressed, and it enhanced GBM growth *via* YTHDF2-MYC-IGFBP3 axis (41). HNRNPA2B1 was highly expressed in multiple types of cancer. HNRNPA2B1-deficient breast cancer cells showed inhibited growth and increased apoptosis rate *via* dampening the phosphorylation of STAT3 (42). Currently, Jiang et al. found that overexpression of HNRNPA2B1 promoted tumor growth and inhibited apoptosis *via* stabilizing ILF3 and AKT3 mRNA transcripts in MM (26). However, our results are inconsistent with a few studies. We found that METTL3 and FTO were significantly downregulated in MM patients, while the expression level of ALKBH5 was similar to normal plasm cells. Xu et al. revealed that FTO was upregulated in MM, especially extramedullary myeloma patients. The overexpression of FTO facilitated cancer progression by stabilizing heat shock transcription factor 1 (HSF1) in a YTHDF2-dependent way (25). In contrast, Bach et al. emphasized the overexpression of METTL3 in MM while no significant differences in the expression of FTO and ALKBH5 (23). The various and specific functions of m6A regulators in different caner types implied that the network of RNA methylation modification is overwhelmingly complicated. Divergences among these studies are probably associated with sampling error, different clinicopathological features, tumor heterogeneity, subclones, and different detection methods. Therefore, what remains to be established is the detailed function of each member in the elaborate m6A methylation network in mediating MM progression and relative mechanisms by which m6A modification alters gene expression at the posttranscriptional level.

Then, to figure out the underlying mechanisms of the risk score, we conducted the differential expression analysis between high- and low-risk groups. Enrichment analysis including overrepresentation analysis (ORA) and GSEA was subsequently conducted. The ORA results showed that oncogenesis-related terms, including mitotic cell cycle phase transition, transcriptional misregulation in cancer, and cell cycle were significantly enriched in upregulated DEG, while immune-related terms, such as response to chemokine, regulation of leukocyte activation, regulation of inflammatory response, regulation of immune effector process, leukocyte proliferation, and migration, were substantially enriched in downregulated DEGs. GSEA results indicated that pathways correlated with tumorigenesis and proteasome inhibitor resistance (MYC targets, unfold protein response, and proteasome) were positively correlated with the high risk score. Since considerable immune-related processes were enriched in downregulated DEGs, we assumed that the m6A risk signature was correlated with the immunosuppressive microenvironment. Thus, we evaluated the immune infiltration level by ESTIMATE algorithm and ssGSEA. As expected, patients in the high-risk group had lower immune score. Sixteen of 28 immune cell subsets, including antitumor immune cells (effector memory CD8 T cell, natural killer cell, natural killer T cell, type 1 T helper cell, and type 17 helper cell) and protumor immune cells (MDSC, regulatory T cell and immature dendritic cell), were significantly reduced in the high-risk group. Moreover, the expression of HLA genes, as cell surface markers for the recognition of immune system, were generally decreased in MM patients with high risk score. Inhibitory immune checkpoint molecules, involved in the immune evasion, PD-L1, and VTCN1, were also significantly downregulated in patients with high risk score. Taken together, we found that the risk score is correlated with the immunosuppressive bone marrow microenvironment from cellular level to molecular level.

The heterogeneity of the tumor immune microenvironment can result in diverse dimensions, such as prognosis and therapeutic response to immunotherapies. However, the underlying mechanism by which tumor cells “reprogrammed” immune microenvironment into the culprit for oncogenesis and drug resistance remains unclear. It was not until 2017 that m6A RNA modification was first elucidated to be involved in the regulation of mammal immune system. METTL3/METTL14-deficient T cells failed to maintain homeostasis and remained in the naive state through IL-7/STAT5/COCS pathway (43). Su et al. reported that forced expression of FTO contributed to the elevated expression of immune checkpoint genes such as PD-L1, PD-L2, and leukocyte immunoglobulin-like receptor subfamily B4 (LILRB4) *via* inhibiting YTHDF2-dependent degradation in AML cell lines (44). Deletion of the demethylase ALKBH5 inhibited the recruitment of immunosuppressive cells (Treg and MDSC) through targeting the key enzyme of lactate transport MCT4/SLC16A3 and decreasing the extracellular lactate concentration, which subsequently resulted in the sensitization of anti-PD-1 therapy in melanoma cells (45). Zhang et al. comprehensively evaluated the m6A modification in gastric cancer and established the m6Ascore to quantify m6A modification patterns for individual patients. They observed that low m6Ascore was characterized by the immune activation with increased efficacy of immune checkpoint blockade treatment, while high m6Ascore was characterized by stroma activation (46). Jin et al. constructed an eight-gene m6A risk signature model in adrenocortical carcinoma to evaluate the prognosis and tumor immune microenvironment (47). Our study also revealed that the m6A-based risk score can accurately predict survival outcomes and evaluate the immune infiltration level.

However, our study does have some limitations. First, the majority of patients included in our study were white; the prognostic value of m6A-related genes remains to be further verified in Asian and African cohorts. Second, given that our results are based on the mRNA expression data, the expression at protein level should be further clarified. Third, some important clinical information, including R-ISS stage, mSMART risk stratification, somatic mutation, and copy number variation, was not available in the GEO database, by which in-depth analysis was inaccessible. Fourth, since the lack of significant clinicopathological information in two validation cohorts, the extrapolation of our nomogram result was not further validated. Thus, experiment verification and clinical investigation with innovative design and orchestrated implement are needed to translate these descriptive results into clinical benefits.

In conclusion, the four m6A regulators (ZC3H13, HNRNPA2B1, HNRNPC, and ZC3H13)-based prognostic risk score can accurately and stably predict the survival of MM patients; and the risk score is tightly associated with the impaired immune infiltration level, which complements current prediction models. The m6A-based prognostic risk score enhances our cognition of immune infiltration and addresses the indispensable role of RNA methylation to cause the heterogeneous tumor microenvironment. Further research is required to understand the overwhelmingly complex immune regulation network in bone marrow microenvironment.

## Data Availability Statement

The public datasets used in this article can be downloaded in the GEO database. The original contributions presented in the study are included in the article/**Supplementary Material.** Further inquiries can be directed to the corresponding authors.

## Author Contributions

RL: conceptualization, data curation, methodology, software, formal analysis, writing—original draft, and visualization. YS: conceptualization, writing—review and editing, supervision, and funding acquisition. JH: conceptualization, formal analysis, methodology, supervision, validation, and writing—review and editing. XW: validation and writing—review and editing. DW: methodology, software, visualization, and writing—review and editing. MZ: conceptualization, methodology, software, validation, visualization, and writing—review and editing. AH: conceptualization, project administration, validation, writing—review and editing, and supervision. JB: conceptualization, project administration, validation, writing—review and editing, and supervision. All authors contributed to the article and approved the submitted version.

## Funding

This work was supported by Natural Science Foundation of Shaanxi Province (Grant/Award Number No. S2020-JC-QN-1401).

## Conflict of Interest

The authors declare that the research was conducted in the absence of any commercial or financial relationships that could be construed as a potential conflict of interest.

## Publisher’s Note

All claims expressed in this article are solely those of the authors and do not necessarily represent those of their affiliated organizations, or those of the publisher, the editors and the reviewers. Any product that may be evaluated in this article, or claim that may be made by its manufacturer, is not guaranteed or endorsed by the publisher.
