# Supplementary material for: Comprehensive Analysis of m6A RNA Methylation Regulators in the Prognosis and Immune Microenvironment of Multiple Myeloma
Source: Front Oncol. 2021 Nov 4;11:731957. doi: 10.3389/fonc.2021.731957 (PMC8599583; doi:10.3389/fonc.2021.731957)
Supplement: Supplementary file 1 [file DataSheet_1.docx]

Supplementary Material

***Additional experiments***

**Methods**

**Subjects**

Bone marrow specimens were obtained from newly diagnosed MM (NDMM) at the department of hematology, The Second Affiliated Hospital of Xi’an Jiaotong University, from 2019 to 2020. The diagnosis, stage and risk status of MM were made in accordance with the National Comprehensive Cancer Network (NCCN) (2020 version 4) and mSMART 3.0. Heathy donor peripheral blood mononuclear cells (BMNCs) samples were used as health control (HC). All samples were isolated using lymphocyte separation liquid to harvest total cellular RNA, then stored at -80°C.

**Cell lines and cell culture**

Human myeloma cell lines MM.1S, RPMI-8226, H929, and human bone marrow stromal cell lines HS-5 were obtained from Professor Jinsong Hu of Xi'an Jiaotong University Health Science Center (Xian, Shaanxi, China). MM cell lines were maintained in RPMI1640 (Hyclone, Logan, UT, USA) with 10% fetal bovine serum (FBS, Biological Industries, Kibbutz Beit Haemek, Israel), penicillin (10,000 U/L, BioSharp, Hefei, China) and streptomycin (100 mg/L, BioSharp, Hefei, China). HS-5 was maintained in high-sugar DMEM (Hyclone, Logan, UT, USA) with 10% FBS, penicillin and streptomycin. All cells were incubated at 37 °C in a humidified atmosphere with 5% CO_2_.

**RT-qPCR**

Total RNA was extracted from mononuclear cells samples using TRIzol reagent (Invitrogen, Germany) and stored at -80 °C until use. RNA purity and concentration were determined by Thermo Scientific Multiskan GO. RNA samples were reversely transcribed into cDNA using a Primescript RT master mix with Oligo dT primers and random primers in accordance with manufacturer's protocols (CWBIO). Then, the qRT-PCR was performed by SYBR Premix Ex Taq™ II (CWBIO) and StepOne Software v2.1 according to manufacturer's instructions. Primers were designed and synthesized by Tsingke (Shanghai, China). And the primer sequences were as shown in Table 1. 2-ΔΔCt value was used to reflect the expression level of each gene. All mRNA levels were normalized to GAPDH.

**Table 1. Primer sequences**

| HNRNPC-F | CCTTACCATCAAACACGATGGC |
| --- | --- |
| HNRNPC-R | ACTTCGAAAAGATTGCCTCCACA |
| YTHDF2-F | TAGCCAACTGCGACACATTC |
| YTHDF2-R | CACGACCTTGACGTTCCTTT |
| HNRNPA2B1-F | AATTCCGCCAACAAACAGCTT |
| HNRNPA2B1-R | ATTGATGGGAGAGTAGTTGAGCC |
| ZC3H13-F | AAAGGAGGTTTCACCAGAAGTG |
| ZC3H13-R | CGCTTCGGAGATTTGCTAGAC |
| GAPDH F | GAAGGTGAAGGTCGGAGTC |
| GAPDH R | GAAGATGGTGATGGGATTTC |

**Results**

HNRNPC, HNRNPA2B1 and YTHDF2 were significantly upregulated, while ZC3H13 was significantly downregulated in MM cell lines H929, RPMI8226 and MM1S compared to normal bone marrow stromal cell lines HS-5 (Figure A-D)

Similarly, the expression levels of HNRNPC, HNRNPA2B1 and YTHDF2 in MM patients were statistically higher than these in normal individuals (P=0.027, P<0.0001, P<0.0001, respectively; Figure E-G). However, the expression level of ZC3H13 in MM patients was lower than it in healthy controls, no significant difference was observed (P=0.16, Figure H). The reason for the inconsistent result may be the insufficient sample size.


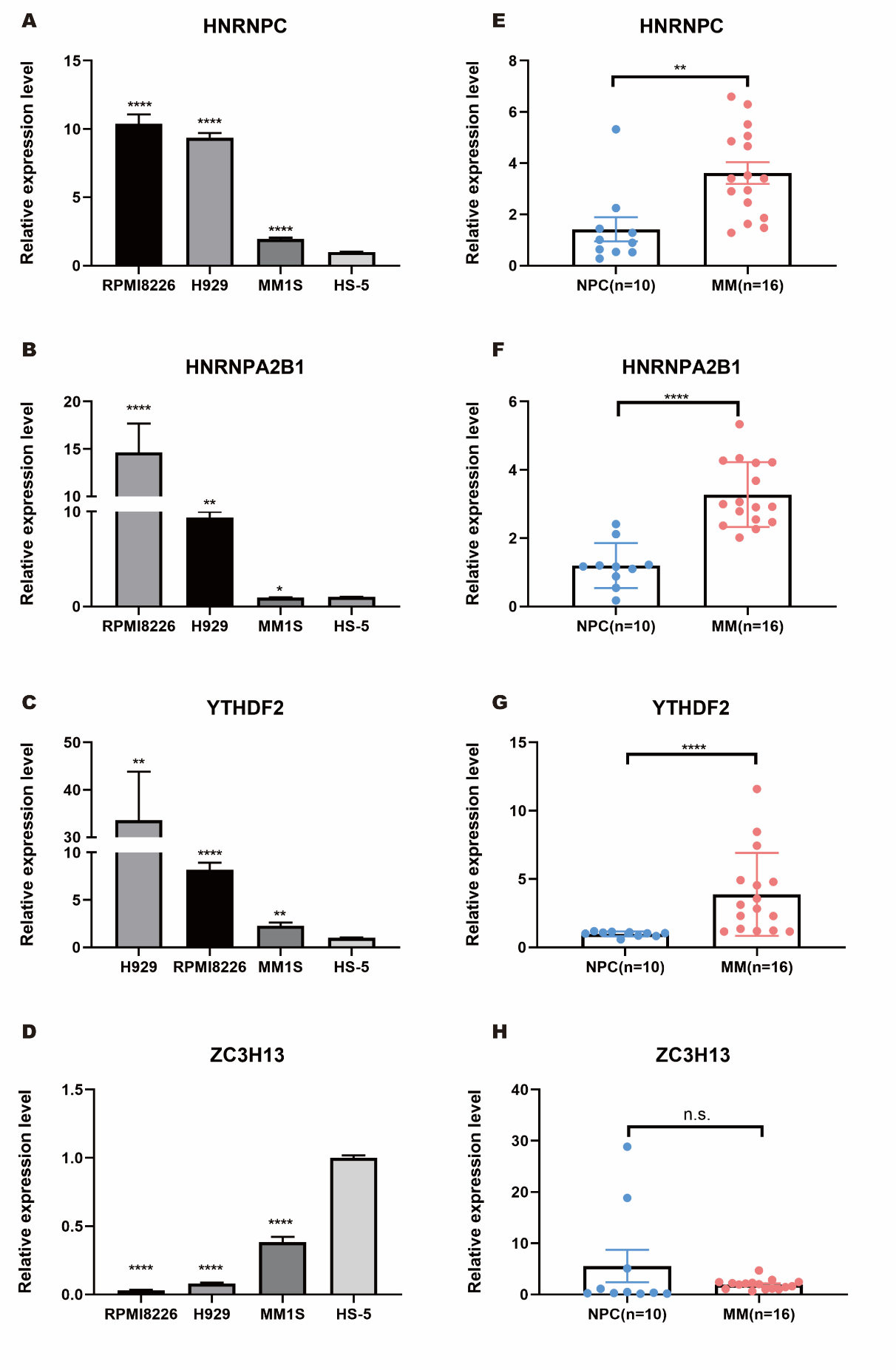


# Supplementary Figures and Tables

**
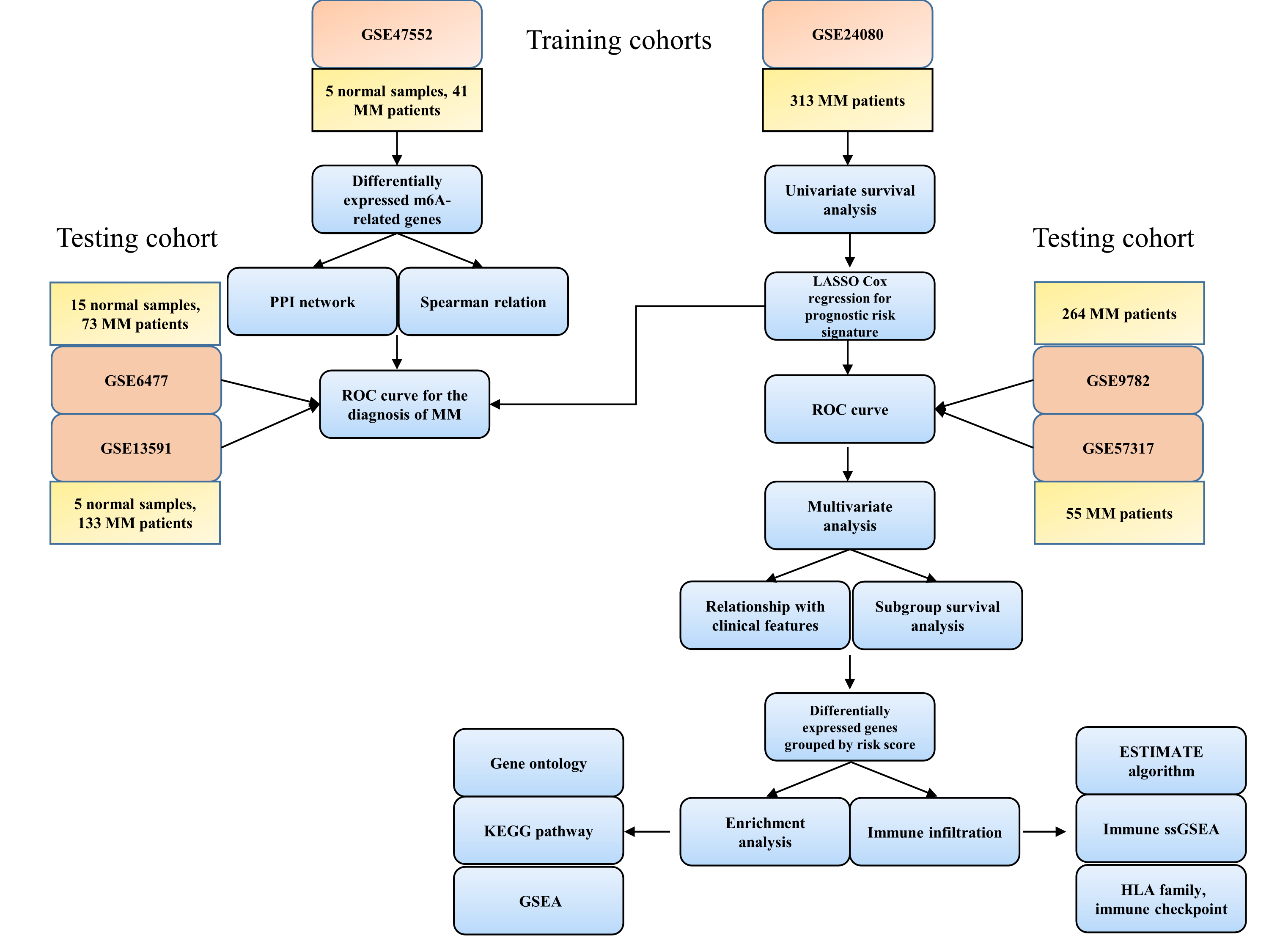
**

**Supplementary Figure 1.The overall design and workflow of this study**

**
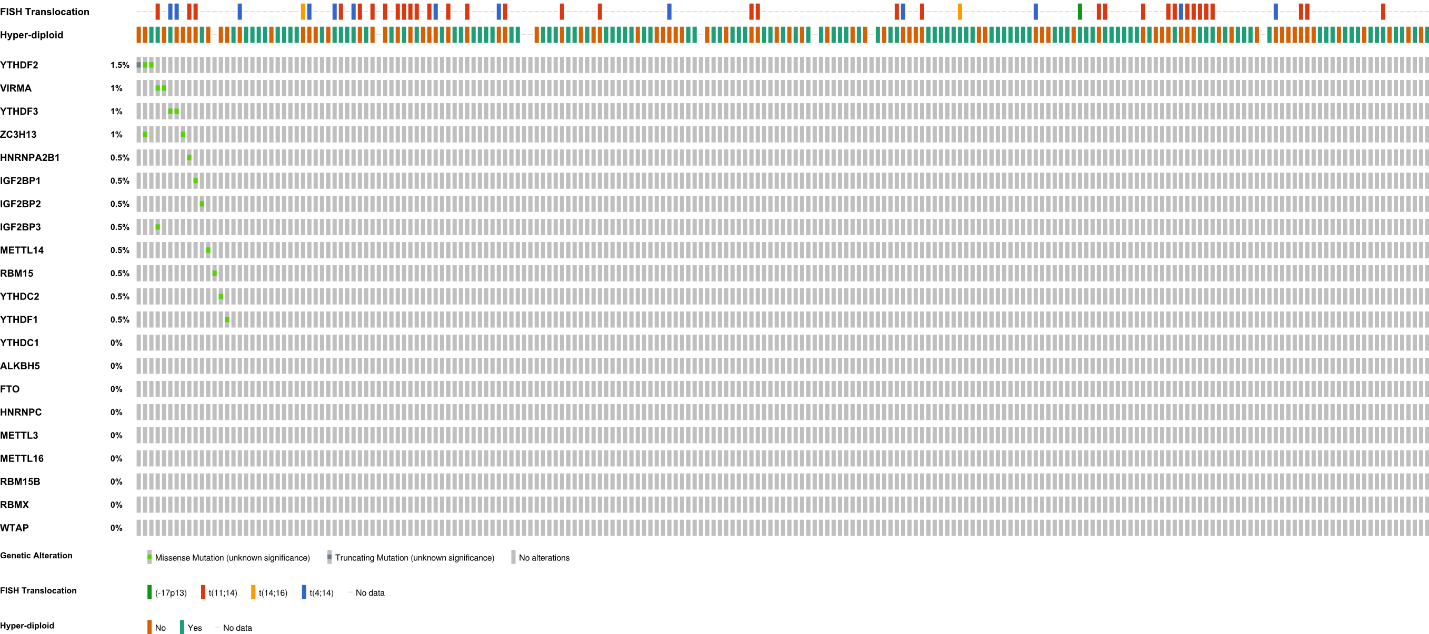
**

**Supplementary Figure 2. The relationship between aberrant expression and genomic instability, including chromosome translocation and hyper-diploid.** We used the online database cBioPortal to determine the mutant frequency of m6A-related genes in 211 multiple myeloma samples, however, no frequent mutation was found.

**
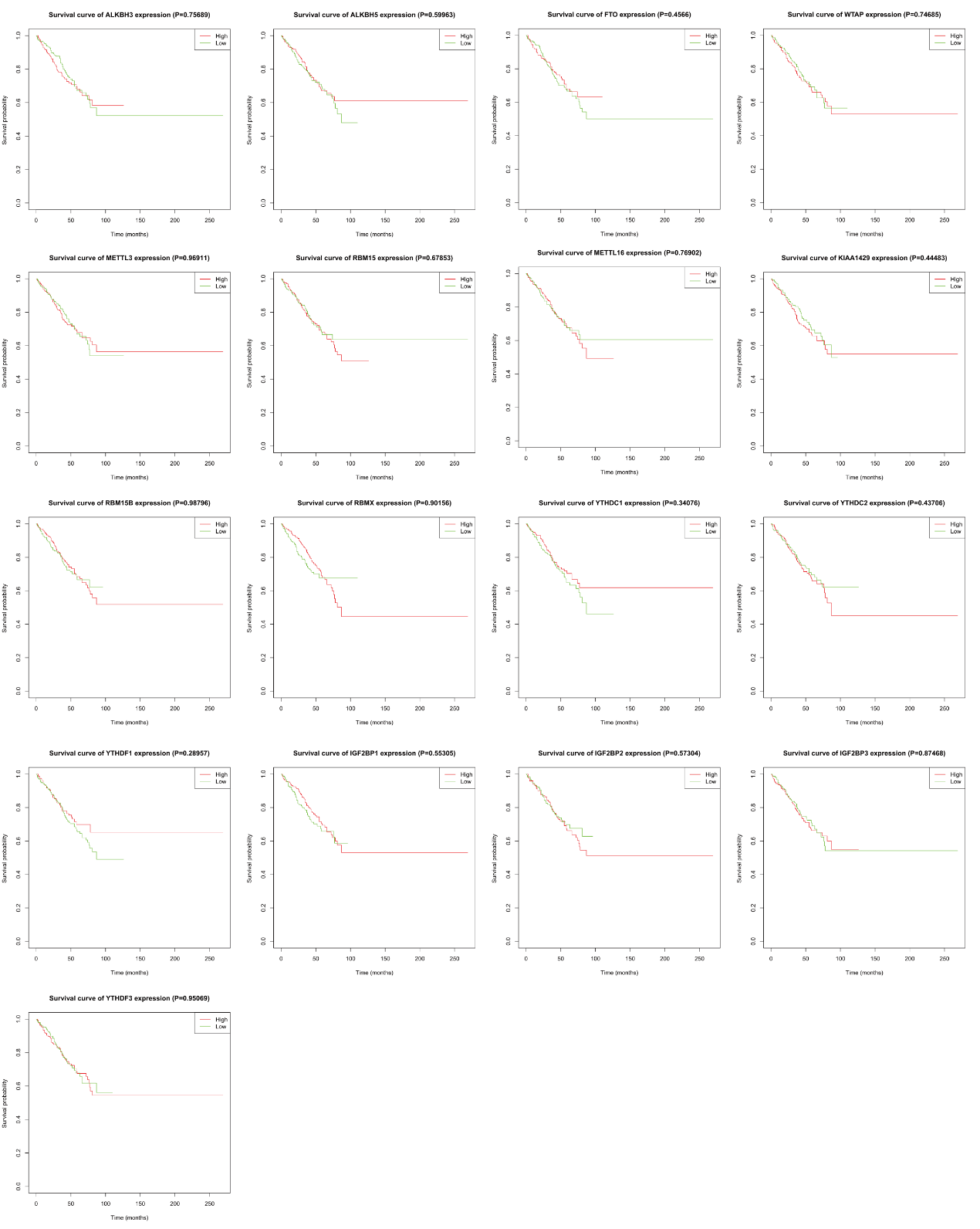
**

**Supplementary Figure 3. Kaplan-Meier survival analysis for m6A-related genes.**


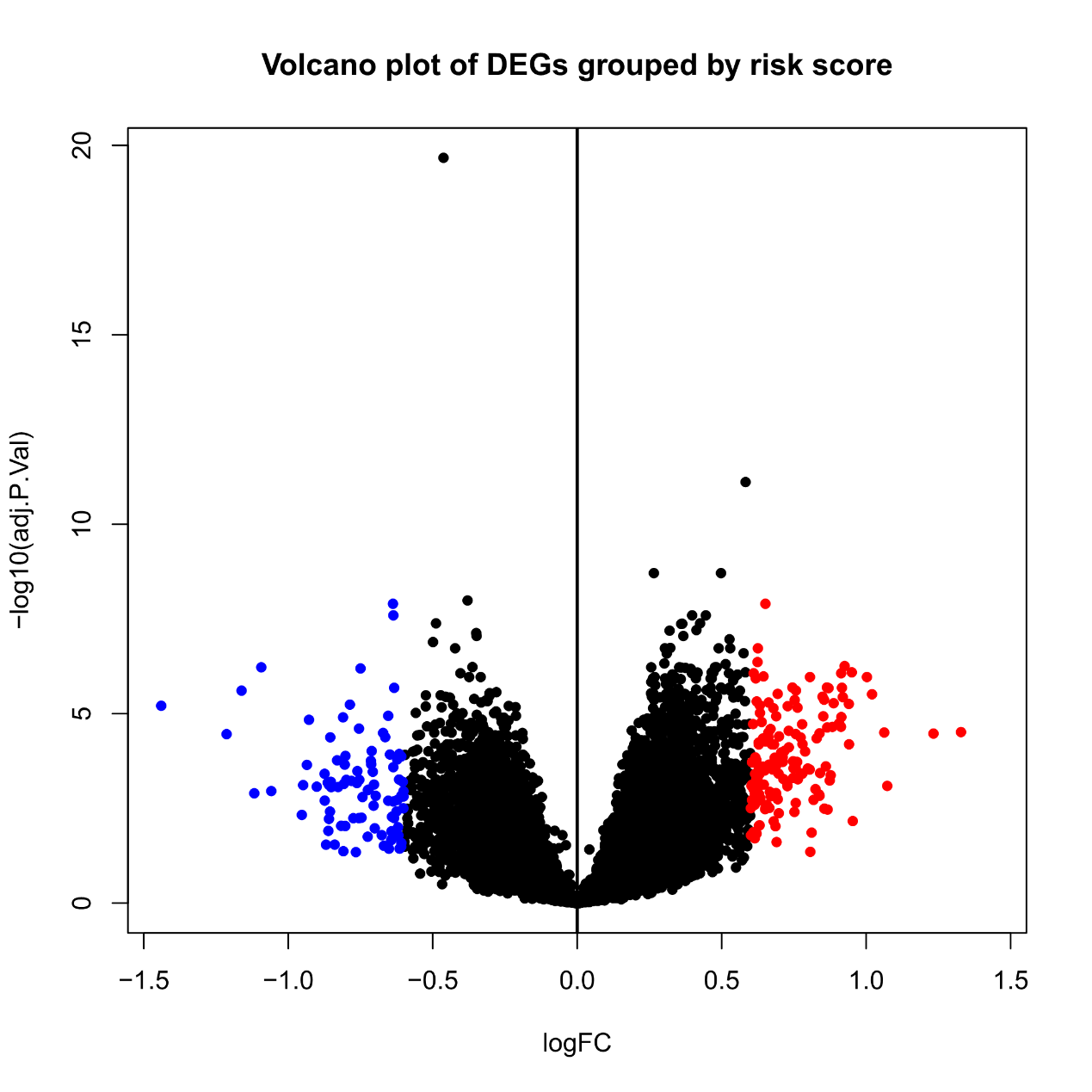


**Supplementary Figure 4. Volcano plot for differentially expressed genes stratified by risk score.** |LogFC|>0.6 and adjusted P value<0.05 was the cut-off criteria.

**Table S1. The information of gene sets used in this study.**

| Accession number | Platform | Number of samples | Country | Years | age(median) | gender(male/female) | race(white/others) | Treatment |
| --- | --- | --- | --- | --- | --- | --- | --- | --- |
| GSE47552 | GPL6244 | 5 NPC, 20 MGUS, 33 SMM and 41 MM | Spain | 2014 | / | / | / | / |
| GSE13591 | GPL96 | 5NPC, 11 MGUS, 133 MM and 9 PCL | Italy | 2009 | / | / | / | / |
| GSE6477 | GPL96 | 15NPC, 22 MGUS, 24 SMM, 73 MM and 28 RRMM | USA | 2007 | / | / | / | / |
| GSE24080 | GPL570 | 554 MM | China | 2010 | 57.75 | 195/118 | 270/43 | TT2: 351  TT3: 214 |
| GSE9782 | GPL96 and GPL97 | 264 MM | USA | 2007 | 61 | 159/105 | 229/35 | DEX:77  Btz: 189 |
| GSE57317 | GPL570 | 55 MM | USA | 2015 | / | / | / | / |

**Table S2. The correlation of four risk genes and clinical characteristics**

| variables | YTHDF2, mean (SD) | | *P* value | HNRNPC, mean (SD) | | *P* value | HNRNPA2B1, mean (SD) | | *P* value | ZC3H13, mean (SD) | | *P* value |  |
| --- | --- | --- | --- | --- | --- | --- | --- | --- | --- | --- | --- | --- | --- |
|  |  |  |  |  |  |  |  |  |  |  |  |  |  |
| Age, years |  |  | 0.220 |  |  | 0.146 |  |  | 0.242 |  |  | 0.348 |  |
| <65 | 11.6718(0.31443) | |  | 10.2541(0.36895) | |  | 10.5382(0.39030) | |  | 9.4182(0.42882) | |  |  |
| >=65 | 11.7052(0.38974) | |  | 10.3141(0.37951) | |  | 10.4734(0.35631) | |  | 9.4729(0.39573) | |  |  |
| Gender |  |  | 0.700 |  |  | **0.019** |  |  | 0.309 |  |  | **0.014** |  |
| female | 11.7026(0.38356) | |  | 10.2045(0.38197) | |  | 10.4957(0.38109) | |  | 9.3573(0.42531) | |  |  |
| male | 11.6937(0.36648) | |  | 10.3079(0.36105) | |  | 10.5384(0.38372) | |  | 9.4766(0.41293) | |  |  |
| Race |  |  | 0.564 |  |  | 0.675 |  |  | 0.694 |  |  | 0.352 |  |
| white | 11.6925(0.37966) | |  | 10.2711(0.37270) | |  | 10.5208(0.39060) | |  | 9.4431(0.41324) | |  |  |
| others | 11.7251(0.32592) | |  | 10.2548(0.37061) | |  | 10.5319(0.33259) | |  | 9.3598(0.46536) | |  |  |
| ISS stage |  |  | **0.045** |  |  | 0.266 |  |  | **0.044** |  |  | 0.106 |  |
| I | 11.6661(0.33939) | |  | 10.2704(0.34648) | |  | 10.4892(0.36052) | |  | 9.4680(0.41989) | |  |  |
| II | 11.7066(0.40567) | |  | 10.2152(0.42793) | |  | 10.5373(0.43813) | |  | 9.3743(0.44538) | |  |  |
| III | 11.7875(0.42817) | |  | 10.3167(0.39157) | |  | 10.6143(0.38487) | |  | 9.3701(0.39227) | |  |  |
| BMPC |  |  | 0.361 |  |  | 0.518 |  |  | **0.022** |  |  | 0.344 |  |
| <46% | 11.6864(0.34063) | |  | 10.2912(0.34700) | |  | 10.4804(0.35906) | |  | 9.4553(0.41615) | |  |  |
| >=46% | 11.7077(0.40273) | |  | 10.2464(0.39516) | |  | 10.5644(0.40183) | |  | 9.4077(0.42575) | |  |  |
| cytogenetic abnormalities |  |  | 0.116 |  |  | **0.009** |  |  | 0.210 |  |  | **0.035** |  |
| yes | 11.7491(0.38145) | |  | 10.3312(0.36061) | |  | 10.5672(0.39261) | |  | 9.3598(0.45651) | |  |  |
| no | 11.6659(0.36437) | |  | 10.2316(0.37440) | |  | 10.4954(0.37507) | |  | 9.4744(0.39323) | |  |  |
| bone lesions |  |  | 0.760 |  |  | 0.231 |  |  | 0.163 |  |  | 0.429 |  |
| yes | 11.7017(0.38267) | |  | 10.2908(0.37122) | |  | 10.5039(0.37900) | |  | 9.4185(0.42481) | |  |  |
| no | 11.6874(0.35224) | |  | 10.2241(0.37095) | |  | 10.5597(0.38924) | |  | 9.4583(0.41375) | |  |  |

**Table S3. Differentially expressed genes of MM patients between high- and low-risk groups**

|  | logFC | AveExpr | t | P.Value | adj.P.Val | B |
| --- | --- | --- | --- | --- | --- | --- |
| TSEN15 | 0.651284 | 9.542244 | 7.235871 | 3.55E-12 | 1.26E-08 | 17.15296 |
| KCNJ2 | -0.63778 | 8.296456 | -7.21792 | 3.97E-12 | 1.26E-08 | 17.04533 |
| CD74 | -0.63647 | 10.27781 | -7.0484 | 1.15E-11 | 2.54E-08 | 16.03815 |
| FTX | 0.625295 | 8.719676 | 6.558028 | 2.23E-10 | 1.88E-07 | 13.22312 |
| REV3L | 0.623989 | 8.081916 | 6.392088 | 5.89E-10 | 4.35E-07 | 12.30492 |
| LOC102724993 | 0.925468 | 11.21218 | 6.333628 | 8.25E-10 | 5.54E-07 | 11.98571 |
| CD79A | -1.09351 | 11.08717 | -6.29187 | 1.05E-09 | 5.98E-07 | 11.75907 |
| BASP1 | -0.75014 | 9.441444 | -6.26625 | 1.21E-09 | 6.41E-07 | 11.62058 |
| GBAP1 | 0.950173 | 6.674923 | 6.211835 | 1.65E-09 | 8.11E-07 | 11.32791 |
| SND1-IT1 | 0.61063 | 8.290668 | 6.187403 | 1.90E-09 | 8.57E-07 | 11.19715 |
| FNTB | 0.913443 | 6.144893 | 6.170748 | 2.09E-09 | 8.57E-07 | 11.10824 |
| ATAD3B///ATAD3A | 0.644738 | 8.32006 | 6.1274 | 2.66E-09 | 1.04E-06 | 10.8777 |
| NOMO3 | 0.805425 | 6.795457 | 6.102978 | 3.05E-09 | 1.08E-06 | 10.74838 |
| DKFZP586B0319 | 1.002717 | 5.728887 | 6.099444 | 3.12E-09 | 1.08E-06 | 10.72969 |
| EZH2 | 0.617862 | 8.52406 | 6.072883 | 3.61E-09 | 1.17E-06 | 10.58957 |
| SLC20A1 | 0.864147 | 8.061965 | 5.947832 | 7.22E-09 | 2.03E-06 | 9.936288 |
| LINC01410 | 0.744726 | 7.902098 | 5.937883 | 7.62E-09 | 2.04E-06 | 9.884771 |
| RNASEH1-AS1 | 0.916177 | 5.87083 | 5.930103 | 7.96E-09 | 2.07E-06 | 9.844537 |
| LINC00607 | -0.63355 | 7.186876 | -5.92776 | 8.06E-09 | 2.08E-06 | 9.832409 |
| SLC26A6 | 0.869584 | 6.613745 | 5.921891 | 8.32E-09 | 2.12E-06 | 9.802112 |
| LOC102724985 | 0.756906 | 6.341404 | 5.885488 | 1.02E-08 | 2.45E-06 | 9.614611 |
| LAPTM5 | -1.1618 | 11.57432 | -5.87742 | 1.06E-08 | 2.47E-06 | 9.573204 |
| GAPDHP62 | 0.693744 | 8.420786 | 5.82515 | 1.41E-08 | 3.00E-06 | 9.305858 |
| NVL | 1.020338 | 7.072477 | 5.817355 | 1.47E-08 | 3.07E-06 | 9.266154 |
| SELPLG | 0.848958 | 10.22947 | 5.77212 | 1.87E-08 | 3.57E-06 | 9.036596 |
| WHSC1 | 0.919081 | 8.435915 | 5.765059 | 1.95E-08 | 3.66E-06 | 9.000895 |
| MSH5-SAPCD1 | 0.854281 | 7.455038 | 5.720819 | 2.47E-08 | 4.27E-06 | 8.778 |
| AGAP9 | 0.752718 | 5.972566 | 5.71519 | 2.54E-08 | 4.35E-06 | 8.749737 |
| POP7 | 0.621213 | 9.283104 | 5.683551 | 3.01E-08 | 4.76E-06 | 8.591301 |
| SNORD50B | 0.662473 | 8.331679 | 5.667674 | 3.27E-08 | 5.08E-06 | 8.512068 |
| GUSBP9///GUSBP3 | 0.887378 | 7.95005 | 5.6545 | 3.51E-08 | 5.29E-06 | 8.446457 |
| NUF2 | 0.94004 | 7.526561 | 5.643869 | 3.71E-08 | 5.55E-06 | 8.3936 |
| BTN1A1 | -0.78689 | 7.992825 | -5.63436 | 3.90E-08 | 5.76E-06 | 8.346416 |
| THBS3 | 0.631957 | 7.153505 | 5.624185 | 4.11E-08 | 6.04E-06 | 8.295949 |
| RGS13 | -1.43979 | 6.018599 | -5.61718 | 4.27E-08 | 6.18E-06 | 8.261249 |
| BCKDHB | 0.728382 | 8.025536 | 5.608648 | 4.46E-08 | 6.34E-06 | 8.219066 |
| ARF1 | 0.762434 | 7.838129 | 5.57856 | 5.23E-08 | 6.94E-06 | 8.070666 |
| SOCS3 | 0.678848 | 7.295895 | 5.569372 | 5.48E-08 | 7.17E-06 | 8.025481 |
| RPUSD2 | 0.632388 | 8.184161 | 5.498297 | 7.93E-08 | 9.56E-06 | 7.677988 |
| CCL8 | -0.65418 | 7.771041 | -5.44921 | 1.02E-07 | 1.14E-05 | 7.440118 |
| ALG1 | 0.688731 | 8.797952 | 5.443439 | 1.05E-07 | 1.17E-05 | 7.412281 |
| SNORA5B | 0.851517 | 7.797299 | 5.442732 | 1.06E-07 | 1.17E-05 | 7.408869 |
| LOC100288637 | 0.914019 | 6.125268 | 5.429843 | 1.13E-07 | 1.23E-05 | 7.346765 |
| MIR548F5///MAB21L1 | -0.81056 | 8.136445 | -5.42464 | 1.16E-07 | 1.25E-05 | 7.321749 |
| BIRC3 | -0.92848 | 10.58669 | -5.38514 | 1.42E-07 | 1.45E-05 | 7.132296 |
| CAPN3 | 0.63826 | 7.299524 | 5.348928 | 1.70E-07 | 1.66E-05 | 6.959664 |
| NOTCH2NL | 0.906007 | 8.631103 | 5.327322 | 1.90E-07 | 1.81E-05 | 6.857115 |
| SRGAP2 | 0.60859 | 5.298784 | 5.316559 | 2.01E-07 | 1.88E-05 | 6.806157 |
| DMAP1 | 0.778381 | 6.430071 | 5.312463 | 2.05E-07 | 1.90E-05 | 6.78679 |
| C1orf112 | 0.912947 | 6.161719 | 5.266939 | 2.57E-07 | 2.22E-05 | 6.57234 |
| SUZ12P1 | 0.883242 | 5.671668 | 5.26545 | 2.59E-07 | 2.23E-05 | 6.565355 |
| PRR5 | 0.865237 | 7.862086 | 5.249446 | 2.81E-07 | 2.31E-05 | 6.490346 |
| CCR2 | -0.75539 | 12.17927 | -5.23184 | 3.07E-07 | 2.48E-05 | 6.408074 |
| NAPB | 0.669091 | 6.368267 | 5.226655 | 3.15E-07 | 2.54E-05 | 6.383861 |
| PKD1P1 | 0.731888 | 6.238408 | 5.199163 | 3.61E-07 | 2.83E-05 | 6.255926 |
| AZGP1 | 1.328161 | 7.981508 | 5.175854 | 4.05E-07 | 3.06E-05 | 6.147899 |
| PTP4A3 | 1.06259 | 9.949108 | 5.166229 | 4.25E-07 | 3.14E-05 | 6.103409 |
| POLR1A | 0.661566 | 6.590995 | 5.163685 | 4.30E-07 | 3.15E-05 | 6.091664 |
| LOC101929272 | -0.67187 | 10.60736 | -5.15596 | 4.47E-07 | 3.23E-05 | 6.056012 |
| EMC3-AS1 | 0.838681 | 5.996923 | 5.149172 | 4.62E-07 | 3.30E-05 | 6.024736 |
| CRIM1 | 1.232867 | 5.860874 | 5.145418 | 4.70E-07 | 3.35E-05 | 6.007455 |
| ATP2A1-AS1 | 0.756542 | 5.978956 | 5.138356 | 4.87E-07 | 3.46E-05 | 5.974967 |
| PPBP | -1.21359 | 9.511054 | -5.13728 | 4.90E-07 | 3.47E-05 | 5.970011 |
| LOC102724562 | 0.698504 | 7.557152 | 5.095524 | 6.01E-07 | 3.97E-05 | 5.778728 |
| ALDH2 | -0.66466 | 11.35697 | -5.08183 | 6.42E-07 | 4.17E-05 | 5.716275 |
| GNG11 | -0.85481 | 7.240746 | -5.07951 | 6.49E-07 | 4.21E-05 | 5.705717 |
| RPL36A | 0.661941 | 6.981098 | 5.07833 | 6.53E-07 | 4.21E-05 | 5.700337 |
| NPIPA5 | 0.773239 | 7.78927 | 5.075656 | 6.61E-07 | 4.24E-05 | 5.688169 |
| PUSL1 | 0.828693 | 7.091094 | 5.059946 | 7.14E-07 | 4.48E-05 | 5.616773 |
| EBLN2 | 0.642956 | 6.338979 | 5.058251 | 7.20E-07 | 4.51E-05 | 5.609083 |
| HSD3B7 | 0.651453 | 6.481145 | 5.011618 | 9.02E-07 | 5.39E-05 | 5.398325 |
| ASPM | 0.632192 | 7.822116 | 4.993899 | 9.82E-07 | 5.76E-05 | 5.318676 |
| PHF19 | 0.627865 | 8.006697 | 4.990476 | 9.98E-07 | 5.81E-05 | 5.303314 |
| POLM | 0.779717 | 7.370426 | 4.964066 | 1.13E-06 | 6.36E-05 | 5.185115 |
| CHEK2 | 0.673208 | 7.23982 | 4.961474 | 1.15E-06 | 6.42E-05 | 5.173542 |
| KIF21B | 0.940694 | 7.379766 | 4.96053 | 1.15E-06 | 6.43E-05 | 5.169329 |
| HIST1H3G | 0.681231 | 5.489085 | 4.956274 | 1.18E-06 | 6.48E-05 | 5.150341 |
| LRRC69 | 0.628542 | 5.617557 | 4.956167 | 1.18E-06 | 6.48E-05 | 5.149863 |
| TACC3 | 0.732255 | 6.042697 | 4.905708 | 1.49E-06 | 7.74E-05 | 4.925817 |
| ANKZF1 | 0.717223 | 8.285191 | 4.841188 | 2.03E-06 | 9.63E-05 | 4.642191 |
| MPEG1 | -0.71137 | 8.698979 | -4.83998 | 2.04E-06 | 9.67E-05 | 4.636918 |
| LOC101060405 | 0.788727 | 6.222681 | 4.828138 | 2.15E-06 | 9.96E-05 | 4.585215 |
| FCHO1 | 0.705926 | 7.025718 | 4.811432 | 2.33E-06 | 0.000106 | 4.512472 |
| LPAR6 | -0.61608 | 7.910994 | -4.78555 | 2.63E-06 | 0.000116 | 4.400176 |
| CNR1 | -0.64824 | 6.503417 | -4.77701 | 2.73E-06 | 0.000119 | 4.363268 |
| LOC100509457 | -0.80223 | 8.210189 | -4.75067 | 3.09E-06 | 0.00013 | 4.2497 |
| LINC01516 | -0.60912 | 7.067394 | -4.74236 | 3.21E-06 | 0.000134 | 4.214011 |
| IL6R | 0.71108 | 10.31766 | 4.725128 | 3.47E-06 | 0.000141 | 4.140118 |
| GPR89B | 0.616329 | 5.420924 | 4.721635 | 3.53E-06 | 0.000143 | 4.125173 |
| ASTE1 | 0.68344 | 7.687456 | 4.715281 | 3.63E-06 | 0.000146 | 4.098005 |
| SHCBP1 | 0.701938 | 8.747341 | 4.702974 | 3.84E-06 | 0.000152 | 4.045478 |
| TLR10 | -0.62305 | 9.47519 | -4.68339 | 4.20E-06 | 0.000162 | 3.962127 |
| LOC101929479 | 0.619629 | 7.491934 | 4.67285 | 4.41E-06 | 0.000166 | 3.917402 |
| SPIC | -0.83106 | 5.775381 | -4.66538 | 4.56E-06 | 0.00017 | 3.885762 |
| KIF11 | 0.612671 | 8.14855 | 4.654927 | 4.78E-06 | 0.000176 | 3.841538 |
| AMIGO2 | -0.7143 | 9.362228 | -4.65383 | 4.81E-06 | 0.000176 | 3.836889 |
| ENPP6 | 0.74707 | 5.788984 | 4.647337 | 4.95E-06 | 0.00018 | 3.80949 |
| NCAPH | 0.761005 | 5.939039 | 4.63345 | 5.27E-06 | 0.000188 | 3.750965 |
| HIST1H1D | 0.711255 | 6.999225 | 4.633332 | 5.27E-06 | 0.000188 | 3.750468 |
| KCNN3 | 0.604626 | 8.729791 | 4.628123 | 5.40E-06 | 0.000191 | 3.728559 |
| CNOT10 | 0.605822 | 8.156904 | 4.625863 | 5.45E-06 | 0.000192 | 3.719055 |
| CDC20 | 0.675537 | 7.572861 | 4.595198 | 6.26E-06 | 0.00021 | 3.59055 |
| SLC46A3 | -0.71325 | 8.802044 | -4.58585 | 6.53E-06 | 0.000217 | 3.551545 |
| CD163 | -0.80475 | 7.039748 | -4.57774 | 6.77E-06 | 0.000222 | 3.51773 |
| AZGP1P1///AZGP1 | 0.663411 | 8.462359 | 4.57727 | 6.79E-06 | 0.000222 | 3.515768 |
| VCAM1 | -0.93563 | 9.140529 | -4.57249 | 6.93E-06 | 0.000225 | 3.495881 |
| CDCA5 | 0.687649 | 7.202425 | 4.550773 | 7.64E-06 | 0.00024 | 3.405703 |
| FBXW4P1 | 0.627615 | 6.410219 | 4.54619 | 7.80E-06 | 0.000244 | 3.386721 |
| ACTG1P4 | 0.86046 | 6.704831 | 4.544195 | 7.87E-06 | 0.000246 | 3.378464 |
| LINC00992 | 0.641889 | 5.491908 | 4.537585 | 8.10E-06 | 0.000249 | 3.351131 |
| CRYBA4 | -0.63672 | 6.116306 | -4.52603 | 8.53E-06 | 0.000259 | 3.303424 |
| HIST1H3F | 0.755764 | 5.112505 | 4.521411 | 8.71E-06 | 0.000263 | 3.284388 |
| ID2B///ID2 | 0.748612 | 7.779937 | 4.504962 | 9.36E-06 | 0.000276 | 3.216727 |
| MGC12488 | 0.797976 | 5.911895 | 4.500307 | 9.56E-06 | 0.00028 | 3.197618 |
| ZNF670 | 0.676698 | 6.875766 | 4.481869 | 1.04E-05 | 0.000298 | 3.122103 |
| FOXM1 | 0.804483 | 5.264727 | 4.481406 | 1.04E-05 | 0.000298 | 3.12021 |
| ASPSCR1 | 0.64906 | 7.024299 | 4.465713 | 1.11E-05 | 0.000315 | 3.056155 |
| LYSMD2 | -0.76091 | 10.04781 | -4.45426 | 1.17E-05 | 0.000328 | 3.009536 |
| HLA-DPA1 | -0.70743 | 8.440599 | -4.44016 | 1.25E-05 | 0.000342 | 2.952294 |
| LOC100996756 | 0.840509 | 7.637078 | 4.415475 | 1.39E-05 | 0.000369 | 2.852423 |
| ID1 | 0.773315 | 6.806271 | 4.402441 | 1.47E-05 | 0.000381 | 2.799898 |
| LOC100131541 | 0.696452 | 4.337492 | 4.402159 | 1.47E-05 | 0.000381 | 2.798761 |
| ARL4A | 0.778894 | 7.503457 | 4.398958 | 1.49E-05 | 0.000385 | 2.785885 |
| FRZB | -0.87436 | 9.895998 | -4.39783 | 1.50E-05 | 0.000386 | 2.781361 |
| SCN9A | 0.623059 | 6.509386 | 4.395779 | 1.51E-05 | 0.000388 | 2.773101 |
| TOMM40L | 0.615604 | 6.741445 | 4.39396 | 1.52E-05 | 0.000388 | 2.765793 |
| SPNS1 | 0.630675 | 8.453288 | 4.391497 | 1.54E-05 | 0.000391 | 2.755901 |
| LOC101930067 | 0.877233 | 5.400574 | 4.369 | 1.70E-05 | 0.000422 | 2.665756 |
| ZNF23 | 0.624067 | 7.638147 | 4.317802 | 2.11E-05 | 0.000494 | 2.462142 |
| SLC12A8 | 0.746938 | 7.380496 | 4.315357 | 2.14E-05 | 0.000497 | 2.452469 |
| FAM209B///FAM209A | 0.713346 | 5.136429 | 4.2958 | 2.32E-05 | 0.00053 | 2.375291 |
| ZNF165 | 0.763746 | 6.530384 | 4.291789 | 2.36E-05 | 0.000536 | 2.3595 |
| C10orf10 | 0.762924 | 7.734189 | 4.291132 | 2.37E-05 | 0.000536 | 2.356917 |
| STAP1 | -0.61658 | 10.58425 | -4.28553 | 2.43E-05 | 0.000546 | 2.334879 |
| PF4V1 | -0.75443 | 5.646095 | -4.2782 | 2.50E-05 | 0.000557 | 2.306113 |
| ZC3H12D | -0.79918 | 8.119634 | -4.27703 | 2.51E-05 | 0.000558 | 2.301504 |
| MIR4271 | 0.619474 | 5.628177 | 4.276412 | 2.52E-05 | 0.000559 | 2.299083 |
| CBR1 | 0.873861 | 7.527171 | 4.261937 | 2.68E-05 | 0.000581 | 2.242388 |
| MLIP | -0.78161 | 9.968915 | -4.25572 | 2.75E-05 | 0.000592 | 2.218094 |
| LOC101060835 | -0.60373 | 9.90625 | -4.23642 | 2.99E-05 | 0.000629 | 2.142844 |
| CD81 | -0.8527 | 9.388662 | -4.23594 | 2.99E-05 | 0.000629 | 2.141004 |
| CPVL | -0.86358 | 8.824136 | -4.22223 | 3.17E-05 | 0.000661 | 2.087745 |
| CCL18 | -0.76249 | 7.542352 | -4.21281 | 3.30E-05 | 0.000679 | 2.051227 |
| IGLJ3///CKAP2 | -0.80785 | 8.476123 | -4.19664 | 3.53E-05 | 0.000715 | 1.988762 |
| RBM10 | 0.645649 | 8.047049 | 4.18318 | 3.73E-05 | 0.000741 | 1.936907 |
| CD27 | -0.70326 | 11.12293 | -4.18308 | 3.73E-05 | 0.000741 | 1.936517 |
| DNAJC17 | 0.632824 | 6.272584 | 4.181301 | 3.76E-05 | 0.000746 | 1.92968 |
| LOC100507006 | 0.604242 | 8.376259 | 4.178523 | 3.80E-05 | 0.000753 | 1.919003 |
| MIR34A | 0.726822 | 6.921052 | 4.176037 | 3.84E-05 | 0.000757 | 1.909449 |
| LAG3 | -0.85919 | 8.121133 | -4.17389 | 3.88E-05 | 0.000761 | 1.901191 |
| KCNA5 | -0.94864 | 8.141994 | -4.17037 | 3.94E-05 | 0.000767 | 1.88769 |
| RPRM | 0.631923 | 7.959217 | 4.164125 | 4.04E-05 | 0.000782 | 1.863759 |
| PTPN20 | 1.072941 | 4.838147 | 4.1538 | 4.22E-05 | 0.000806 | 1.824249 |
| RRM2 | 0.603217 | 10.57913 | 4.152844 | 4.23E-05 | 0.000808 | 1.820593 |
| PKP2 | 0.72722 | 6.394737 | 4.14965 | 4.29E-05 | 0.000817 | 1.808392 |
| IGHV4-31 | -0.9016 | 11.51295 | -4.13715 | 4.52E-05 | 0.000847 | 1.760699 |
| LOC100293211 | -0.82757 | 8.031626 | -4.12976 | 4.66E-05 | 0.000864 | 1.732577 |
| IGHA1 | -0.85151 | 11.10897 | -4.12681 | 4.71E-05 | 0.00087 | 1.721378 |
| KIFC1 | 0.609799 | 6.822855 | 4.119831 | 4.85E-05 | 0.000885 | 1.694875 |
| HIST1H2AJ | 0.619643 | 7.57213 | 4.088026 | 5.53E-05 | 0.000979 | 1.574608 |
| TREML2 | 0.824628 | 6.310612 | 4.086862 | 5.55E-05 | 0.000981 | 1.570223 |
| LOC101927266 | 0.610793 | 5.427165 | 4.083463 | 5.63E-05 | 0.000991 | 1.557422 |
| APOL3 | -0.72202 | 9.314916 | -4.07717 | 5.78E-05 | 0.001008 | 1.533754 |
| ABCG2 | -0.72467 | 10.04668 | -4.06694 | 6.02E-05 | 0.001035 | 1.495315 |
| CCND1 | -1.05903 | 9.57903 | -4.04386 | 6.62E-05 | 0.001103 | 1.408966 |
| LSP1 | -0.60142 | 10.35528 | -4.04068 | 6.70E-05 | 0.001113 | 1.397132 |
| TRIM47 | 0.66667 | 7.635452 | 4.026332 | 7.10E-05 | 0.001158 | 1.3437 |
| LOC105379362 | 0.688048 | 7.565248 | 4.002964 | 7.81E-05 | 0.001248 | 1.257064 |
| KIF4A | 0.614135 | 7.336315 | 4.002147 | 7.83E-05 | 0.001251 | 1.254042 |
| CTSW | -1.11778 | 6.953837 | -3.99791 | 7.97E-05 | 0.001265 | 1.238401 |
| MIR142 | 0.629219 | 7.13879 | 3.981918 | 8.49E-05 | 0.001327 | 1.179423 |
| HIST1H3D///HIST1H2AD | 0.836834 | 6.680644 | 3.975084 | 8.73E-05 | 0.001352 | 1.154293 |
| C1orf106 | 0.688089 | 8.709008 | 3.973575 | 8.78E-05 | 0.001355 | 1.148749 |
| CXCL12 | -0.60855 | 9.00457 | -3.95018 | 9.64E-05 | 0.001454 | 1.063045 |
| GAGE12F | 0.838601 | 5.387578 | 3.946223 | 9.79E-05 | 0.00147 | 1.048588 |
| CHSY3 | -0.69738 | 9.569315 | -3.9439 | 9.88E-05 | 0.001474 | 1.040107 |
| LOC100996735 | 0.673497 | 6.436359 | 3.942538 | 9.94E-05 | 0.00148 | 1.03514 |
| POLE2 | 0.629481 | 7.070316 | 3.934663 | 0.000103 | 0.001515 | 1.006442 |
| GRB14 | 0.669688 | 5.584744 | 3.934309 | 0.000103 | 0.001516 | 1.005153 |
| APOE | -0.60103 | 8.264486 | -3.92902 | 0.000105 | 0.001532 | 0.985908 |
| TAGLN2 | 0.62432 | 9.753393 | 3.920583 | 0.000108 | 0.001572 | 0.955262 |
| TIMD4 | -0.74307 | 8.595235 | -3.91696 | 0.00011 | 0.001587 | 0.942118 |
| RRN3P2 | 0.633348 | 6.431684 | 3.899375 | 0.000118 | 0.001668 | 0.878484 |
| MYBL2 | 0.693918 | 6.739995 | 3.868618 | 0.000133 | 0.001816 | 0.767807 |
| BMP4 | 0.81784 | 5.446528 | 3.855028 | 0.00014 | 0.001887 | 0.719159 |
| CD5L | -0.62221 | 8.377684 | -3.85396 | 0.000141 | 0.001892 | 0.715346 |
| KIF20A | 0.604718 | 7.548892 | 3.852591 | 0.000142 | 0.0019 | 0.710451 |
| CENPE | 0.630805 | 6.674937 | 3.84434 | 0.000146 | 0.001946 | 0.681005 |
| IGHG1 | -0.87392 | 10.7559 | -3.83992 | 0.000149 | 0.001968 | 0.66525 |
| PKIA-AS1 | -0.654 | 4.592392 | -3.8376 | 0.00015 | 0.001981 | 0.656993 |
| KCNMB2 | -0.6367 | 8.152885 | -3.82184 | 0.00016 | 0.00208 | 0.600994 |
| PITPNM1 | 0.60975 | 6.842597 | 3.798536 | 0.000175 | 0.002244 | 0.518593 |
| GAGE12D | 0.756352 | 6.764555 | 3.793539 | 0.000178 | 0.002272 | 0.500984 |
| LOC100134822 | 0.614162 | 5.06249 | 3.754514 | 0.000207 | 0.002531 | 0.364171 |
| WHRN | 0.656478 | 7.058309 | 3.75051 | 0.00021 | 0.002559 | 0.350206 |
| VPREB3 | -0.70484 | 8.679462 | -3.73401 | 0.000224 | 0.00268 | 0.29279 |
| HIST1H2AM | 0.664813 | 6.88144 | 3.687522 | 0.000267 | 0.003077 | 0.132322 |
| ZMYND19 | 0.651514 | 7.763543 | 3.685315 | 0.000269 | 0.003097 | 0.124749 |
| SAC3D1 | 0.600736 | 8.654613 | 3.680368 | 0.000274 | 0.003123 | 0.107787 |
| SYK | -0.60158 | 7.781099 | -3.67874 | 0.000276 | 0.003134 | 0.102193 |
| CCND2 | 0.855153 | 8.747427 | 3.669908 | 0.000285 | 0.003203 | 0.071994 |
| PTPRM | 0.650305 | 7.041358 | 3.65557 | 0.000301 | 0.003313 | 0.023084 |
| CRNDE | 0.866587 | 9.072695 | 3.647767 | 0.000309 | 0.003389 | -0.00346 |
| MDK | -0.62544 | 8.951177 | -3.60855 | 0.000358 | 0.003819 | -0.13609 |
| PRG2 | -0.85567 | 8.275323 | -3.60778 | 0.000359 | 0.003828 | -0.13868 |
| GAGE12D | 0.751716 | 5.886233 | 3.599836 | 0.00037 | 0.003925 | -0.16537 |
| HIST1H3F | 0.697702 | 7.839824 | 3.568958 | 0.000414 | 0.004252 | -0.26864 |
| RRAS2 | -0.95317 | 7.757874 | -3.53398 | 0.000471 | 0.004688 | -0.38461 |
| GLIS3 | -0.64128 | 6.973513 | -3.49027 | 0.000551 | 0.005249 | -0.52808 |
| MS4A1 | -0.74648 | 8.974299 | -3.4687 | 0.000596 | 0.00556 | -0.59827 |
| RNASE3 | -0.6338 | 7.093016 | -3.46031 | 0.000614 | 0.005677 | -0.62548 |
| C1QC | -0.75556 | 8.689039 | -3.4582 | 0.000619 | 0.005704 | -0.63229 |
| NOL4 | -0.7749 | 7.647321 | -3.45693 | 0.000621 | 0.005725 | -0.6364 |
| IGHM | -0.85933 | 9.326635 | -3.43917 | 0.000662 | 0.005997 | -0.69371 |
| KCNS3 | 0.953606 | 9.123869 | 3.389937 | 0.000788 | 0.006828 | -0.85114 |
| GAGE12D | 0.680068 | 6.809104 | 3.384887 | 0.000802 | 0.006924 | -0.86717 |
| CDK5 | 0.631076 | 7.806871 | 3.293316 | 0.001103 | 0.008821 | -1.15398 |
| LOC101929141 | 0.629189 | 7.661956 | 3.285442 | 0.001133 | 0.009007 | -1.1783 |
| C1QA | -0.81715 | 7.57295 | -3.28017 | 0.001154 | 0.009123 | -1.19457 |
| CD19 | -0.80258 | 6.954061 | -3.27594 | 0.00117 | 0.009229 | -1.20759 |
| CFI | 0.686088 | 6.602333 | 3.270107 | 0.001194 | 0.009362 | -1.22551 |
| HMOX1 | -0.62037 | 9.156193 | -3.2445 | 0.001303 | 0.009962 | -1.30387 |
| IGHV3-23 | -0.70083 | 11.03586 | -3.22124 | 0.00141 | 0.010596 | -1.37459 |
| IGHV3-23 | -0.86139 | 9.969843 | -3.15501 | 0.00176 | 0.012375 | -1.57324 |
| IGHM///IGHG1 | -0.64288 | 7.56875 | -3.14203 | 0.001837 | 0.012718 | -1.61176 |
| CTAG1A///CTAG1B | 0.609377 | 5.408702 | 3.120546 | 0.001972 | 0.013364 | -1.67511 |
| FGFR3 | 0.811308 | 6.98607 | 3.106933 | 0.002062 | 0.013795 | -1.71506 |
| VWDE | 0.620847 | 5.184012 | 3.083594 | 0.002226 | 0.014572 | -1.78316 |
| GBA3 | -0.6765 | 9.7749 | -3.04133 | 0.002553 | 0.016072 | -1.90524 |
| PARP15 | 0.601254 | 5.637694 | 3.038738 | 0.002575 | 0.016175 | -1.91269 |
| SPINT2 | -0.61891 | 10.49046 | -3.0337 | 0.002617 | 0.016361 | -1.92712 |
| IGKV1OR2-108 | -0.72515 | 12.24762 | -2.99882 | 0.002926 | 0.017677 | -2.02645 |
| IGLJ3 | -0.63906 | 11.13609 | -2.97391 | 0.003167 | 0.018785 | -2.09674 |
| MATR3 | 0.614415 | 7.74098 | 2.957604 | 0.003335 | 0.019539 | -2.14243 |
| IGLJ3///IGLV@///IGLC1 | -0.64384 | 12.14928 | -2.90863 | 0.003888 | 0.021769 | -2.27828 |
| TSPAN7 | 0.690009 | 9.452254 | 2.858017 | 0.004547 | 0.024462 | -2.41639 |
| QPCT | -0.60871 | 12.12158 | -2.83334 | 0.004903 | 0.02584 | -2.4829 |
| LOC102725526 | -0.83934 | 10.49439 | -2.78618 | 0.005657 | 0.028612 | -2.60849 |
| ISL2 | -0.86941 | 8.42047 | -2.78482 | 0.00568 | 0.028707 | -2.61208 |
| PF4 | -0.66932 | 7.5254 | -2.75504 | 0.00621 | 0.03062 | -2.69033 |
| IGLL5 | -0.60947 | 11.37215 | -2.72764 | 0.006737 | 0.032411 | -2.7616 |
| CCR5 | -0.61483 | 8.157222 | -2.67112 | 0.007952 | 0.036509 | -2.90647 |
| HLA-DRA | -0.65179 | 8.889016 | -2.66886 | 0.008005 | 0.036655 | -2.91221 |
| EDNRB | -0.8095 | 9.860904 | -2.59604 | 0.009872 | 0.042655 | -3.09446 |
| S100A4 | 0.80676 | 9.060982 | 2.578255 | 0.010384 | 0.044236 | -3.13823 |
| IFI44L | -0.76602 | 6.591131 | -2.56925 | 0.010652 | 0.045126 | -3.16029 |

**Table S4. The results of GSEA grouped by risk score**

| Hallmark | | | | | | |
| --- | --- | --- | --- | --- | --- | --- |
| NAME | SIZE | ES | NES | NOM p-val | FDR q-val | FWER p-val |
| HALLMARK_MYC_TARGETS_V1 | 161 | 0.6683556 | 2.3038018 | 0 | 3.85E-04 | 0.001 |
| HALLMARK_MYC_TARGETS_V2 | 50 | 0.6740854 | 1.9187313 | 0.0018797 | 0.0191193 | 0.049 |
| HALLMARK_E2F_TARGETS | 155 | 0.691939 | 1.794064 | 0.018622 | 0.0292227 | 0.11 |
| HALLMARK_UNFOLDED_PROTEIN_RESPONSE | 96 | 0.4395706 | 1.7846229 | 0.0238095 | 0.0233702 | 0.117 |
| HALLMARK_OXIDATIVE_PHOSPHORYLATION | 167 | 0.4111812 | 1.6418428 | 0.0956023 | 0.0454227 | 0.246 |
| KEGG | | | | | | |
| KEGG_SPLICEOSOME | 77 | 0.6003842 | 2.3538845 | 0 | 0 | 0 |
| KEGG_PROTEASOME | 38 | 0.5942749 | 1.8810173 | 0.0251938 | 0.0392277 | 0.111 |
| Gene Ontology - Biological Process | | | | | | |
| GOBP_RIBONUCLEOPROTEIN_COMPLEX_BIOGENESIS | 255 | 0.5415534 | 2.3366175 | 0 | 0.0097571 | 0.012 |
| GOBP_SPLICEOSOMAL_COMPLEX_ASSEMBLY | 32 | 0.6452309 | 2.323662 | 0.002004 | 0.0057716 | 0.012 |
| GOBP_RIBONUCLEOPROTEIN_COMPLEX_SUBUNIT_ORGANIZATION | 120 | 0.5099037 | 2.2316017 | 0 | 0.0121821 | 0.029 |
| GOBP_RIBOSOME_BIOGENESIS | 176 | 0.5278083 | 2.2189195 | 0 | 0.0104709 | 0.034 |
| GOBP_TRNA_TRANSPORT | 31 | 0.6229442 | 2.11397 | 0 | 0.026291 | 0.106 |
| GOBP_DNA_TEMPLATED_TRANSCRIPTION_TERMINATION | 50 | 0.5710488 | 2.085957 | 0.002045 | 0.0294916 | 0.14 |
| GOBP_NCRNA_EXPORT_FROM_NUCLEUS | 32 | 0.6048552 | 2.081844 | 0.0038986 | 0.0263603 | 0.143 |
| GOBP_RRNA_METABOLIC_PROCESS | 134 | 0.5237259 | 2.0680408 | 0.0020534 | 0.0270414 | 0.166 |
| GOBP_NCRNA_PROCESSING | 208 | 0.4895141 | 2.0584722 | 0.004158 | 0.0268537 | 0.186 |
| GOBP_MATURATION_OF_5_8S_RRNA | 21 | 0.6557854 | 2.0421128 | 0.002045 | 0.0283446 | 0.206 |
| GOBP_MITOCHONDRIAL_GENE_EXPRESSION | 90 | 0.5087335 | 2.030411 | 0.0061728 | 0.0290203 | 0.224 |
| GOBP_ANAPHASE_PROMOTING_COMPLEX_DEPENDENT_CATABOLIC_PROCESS | 65 | 0.6135747 | 1.9965316 | 0.0061728 | 0.0366395 | 0.274 |
| GOBP_POSTREPLICATION_REPAIR | 37 | 0.6664686 | 1.9963877 | 0.0059524 | 0.0338636 | 0.274 |
| GOBP_TRANSLATIONAL_TERMINATION | 63 | 0.5007636 | 1.9899206 | 0.0219124 | 0.0333349 | 0.285 |
| GOBP_RNA_SPLICING_VIA_TRANSESTERIFICATION_REACTIONS | 217 | 0.4115799 | 1.9885695 | 0.0081301 | 0.0315795 | 0.29 |
| GOBP_POSITIVE_REGULATION_OF_MRNA_METABOLIC_PROCESS | 47 | 0.4734611 | 1.9742773 | 0.0020704 | 0.0333223 | 0.32 |
| GOBP_TRANSLESION_SYNTHESIS | 31 | 0.6611438 | 1.964359 | 0.0060362 | 0.0342474 | 0.333 |
| GOBP_MITOCHONDRIAL_TRANSLATION | 75 | 0.5252659 | 1.962555 | 0.0206186 | 0.0327408 | 0.334 |
| GOBP_HISTONE_MRNA_METABOLIC_PROCESS | 16 | 0.7100452 | 1.946475 | 0.0040161 | 0.0353146 | 0.359 |
| GOBP_NCRNA_METABOLIC_PROCESS | 241 | 0.4327221 | 1.9319004 | 0.0062241 | 0.0376143 | 0.391 |
| GOBP_SPLICEOSOMAL_SNRNP_ASSEMBLY | 30 | 0.6857373 | 1.9303899 | 0.0104167 | 0.0363327 | 0.392 |
| GOBP_TERMINATION_OF_RNA_POLYMERASE_I_TRANSCRIPTION | 21 | 0.5713681 | 1.9287524 | 0.0140562 | 0.035288 | 0.397 |
| GOBP_MITOCHONDRIAL_TRANSLATIONAL_TERMINATION | 53 | 0.53644 | 1.9269398 | 0.0305499 | 0.0341864 | 0.404 |
| GOBP_TRANSCRIPTION_INITIATION_FROM_RNA_POLYMERASE_I_PROMOTER | 25 | 0.5409365 | 1.926086 | 0.0160643 | 0.0329386 | 0.408 |
| GOBP_REGULATION_OF_MRNA_CATABOLIC_PROCESS | 141 | 0.3535128 | 1.9043934 | 0.0063025 | 0.0374086 | 0.471 |
| GOBP_TRANSLATIONAL_ELONGATION | 74 | 0.4265571 | 1.8924518 | 0.0240481 | 0.0396409 | 0.511 |
| GOBP_POSITIVE_REGULATION_OF_MRNA_CATABOLIC_PROCESS | 27 | 0.5130729 | 1.886126 | 0.0099404 | 0.0402969 | 0.536 |
| GOBP_RNA_EXPORT_FROM_NUCLEUS | 87 | 0.425751 | 1.8829042 | 0.0220441 | 0.0396629 | 0.542 |
| GOBP_ERROR_PRONE_TRANSLESION_SYNTHESIS | 17 | 0.7337502 | 1.8778697 | 0.0081466 | 0.0399274 | 0.556 |
| GOBP_NUCLEAR_TRANSCRIBED_MRNA_CATABOLIC_PROCESS_DEADENYLATION_DEPENDENT_DECAY | 47 | 0.4249079 | 1.8729395 | 0.0041408 | 0.0404536 | 0.57 |
| GOBP_TERMINATION_OF_RNA_POLYMERASE_II_TRANSCRIPTION | 25 | 0.6228651 | 1.8644289 | 0.0082305 | 0.0418997 | 0.591 |
| GOBP_TELOMERE_MAINTENANCE_VIA_TELOMERE_LENGTHENING | 49 | 0.4586034 | 1.8630642 | 0.0081633 | 0.041085 | 0.595 |
| GOBP_REGULATION_OF_MRNA_METABOLIC_PROCESS | 219 | 0.3198263 | 1.8587744 | 0.0081633 | 0.0412607 | 0.61 |
| GOBP_PROTEASOMAL_UBIQUITIN_INDEPENDENT_PROTEIN_CATABOLIC_PROCESS | 18 | 0.6436768 | 1.8584692 | 0.00611 | 0.0401097 | 0.611 |
| GOBP_TRNA_METABOLIC_PROCESS | 77 | 0.4867972 | 1.8577884 | 0.0083333 | 0.039059 | 0.613 |
| GOBP_CYTOKINETIC_PROCESS | 23 | 0.7030087 | 1.8544738 | 0.0040568 | 0.0389503 | 0.627 |
| GOBP_G0_TO_G1_TRANSITION | 34 | 0.5693262 | 1.8496776 | 0.0061602 | 0.0394827 | 0.643 |
| GOBP_NUCLEAR_TRANSCRIBED_MRNA_CATABOLIC_PROCESS_EXONUCLEOLYTIC | 27 | 0.5581349 | 1.8490407 | 0.0183299 | 0.0386323 | 0.645 |
| GOBP_RIBOSOMAL_LARGE_SUBUNIT_BIOGENESIS | 36 | 0.5699912 | 1.8450533 | 0.0125261 | 0.038824 | 0.661 |
| GOBP_TRNA_PROCESSING | 63 | 0.4852267 | 1.8388599 | 0.0122449 | 0.0397106 | 0.679 |
| GOBP_RNA_SPLICING | 267 | 0.3458973 | 1.8150808 | 0.0184426 | 0.0469226 | 0.767 |
| GOBP_POSITIVE_REGULATION_OF_MRNA_PROCESSING | 20 | 0.5726915 | 1.803215 | 0.01 | 0.0490572 | 0.797 |
